# Mobile Cancer Screening Programs: A Systematic Review of Implementation Challenges and Population Access

**DOI:** 10.3390/ijerph23040465

**Published:** 2026-04-04

**Authors:** Safa ElKefi, Roberta Scheinmann, Alicia K. Matthews, Erica Phillips

**Affiliations:** 1 School of System Science & Industrial Engineering, The Thomas J. Watson College of Engineering and Applied Science, Binghamton University, Binghamton, NY 13902, USA; 2 School of Nursing, Columbia University, New York, NY 10032, USA; 3 College of Nursing, Rush University, Chicago, IL 60612, USA; 4 Division of General Internal Medicine, Weill Cornell Medical College, Cornell University, New York, NY 10021, USA; erp2001@med.cornell.edu; 5 Cancer Prevention and Control Program, Sandra and Edward Meyer Cancer Center at Weill Cornell Medical College, Cornell University, New York, NY 10021, USA

**Keywords:** mobile unit, mobile clinic, cancer screening, community health, cancer prevention

## Abstract

**Highlights:**

**Public health relevance—How does this work relate to a public health issue?**

- Mobile screening programs address cancer screening disparities driven by structural barriers and geographic locations.
- This review summarizes global evidence on the design and implementation of mobile screening programs and units.

**Public health significance—Why is this work of significance to public health?**

- Across the 64 included studies, mobile screening units were linked to better screening outcomes, especially in underserved communities.
- This study identified the common bottlenecks in the system, limiting the impact and potential of mobile screening units.

**Public health implications—What are the key implications or messages for practitioners, policy makers and/or researchers in public health?**

- Mobile screening units are effective when (1) embedded in community-engaged deployment (sites + outreach) and (2) linked to clear referral pathways.
- Future research should work on stronger comparative designs and standardized reporting of follow-up outcomes to evaluate the equity impact and sustainability of these interventions.

**Abstract:**

Objective: This review aimed to synthesize evidence on the design characteristics, implementation considerations, and operational challenges of mobile cancer screening units. Methods: A PRISMA-guided review was conducted. Data extracted included screening type, target population, program characteristics, mobile unit features, and reported implementation barriers. Study quality was assessed using the Mixed Methods Appraisal Tool (MMAT). Results: Sixty-four articles published across 13 countries met the inclusion criteria. Most interventions focused on breast cancer screening (n = 37), followed by lung (n = 11), cervical (n = 6), colorectal, skin, prostate, and multi-cancer screening programs. MSUs were most frequently deployed in urban areas (21 urban/18 rural/17 both). Several comparative studies (fixed programs vs. MSUs) reported higher screening uptake in mobile programs, although findings varied substantially by setting, population, and study design. However, adherence and clinical outcomes varied, often reflecting baseline socioeconomic differences in the populations served. Common implementation barriers included follow-up coordination challenges, program costs, equipment and space limitations, and gaps in referral and reimbursement systems. Conclusions: These findings highlight the potential of mobile screening units as public health strategies to expand access to cancer screening while underscoring the need for stronger implementation frameworks and long-term evaluation of program outcomes.

## 1. Introduction

Cancer is a leading cause of morbidity and mortality worldwide and imposes a heavy burden on healthcare systems. According to the World Health Organization (WHO), in 2022, there were an estimated 20 million new cases of cancer and 9.7 million cancer deaths. About 20% of the population will develop cancer in their lifetime. Global cancer statistics show that lung cancer is the leading incident cancer worldwide (12.4%), ahead of female breast (11.6%), colorectal (9.6%), prostate (7.3%), and stomach cancers (4.9%). The WHO estimates that, by 2050, worldwide cancer diagnoses will increase by 77% [1]. In high-income countries, screening enables many cancers to be detected at a stage when treatment may still be feasible. Therefore, both WHO and the Centers for Disease Control and Prevention recommend screening where feasible [1]. However, studies of cancer screening types, including lung, cervical, and colorectal cancers, show multiple disparities in receipt of screening. These disparities exist with respect to race/ethnicity, socioeconomic/poverty status, and urban/rural differences [2].

Most healthcare settings offer hospital-based or fixed-site screening programs. However, many barriers to accessing fixed-site screening services exist. For example, (1) individuals living in rural areas or with limited transportation options can have trouble reaching screening centers; (2) lack of insurance coverage for certain procedures can marginalize vulnerable patients, leaving them without access to screening; and (3) even with insurance, out-of-pocket costs associated with screening and follow-up care can be a barrier for low-income individuals [3,4].

Mobile screening units (MSUs) and clinics provide cancer screening services outside traditional fixed-site facilities. MSUs may include vans, recreational vehicles, or other mobile clinics, reducing structural barriers related to transportation, working hours, and geographic access to fixed-site screening programs. A review by Greenwald et al. for research published up to July 2015 showed that MSUs for mammography are successful at reaching otherwise hard-to-reach populations that are at risk of developing breast, colon, and cervical cancer [5]. While the focus on these 3 types of cancer is important, research is scarce on MSUs for other types of cancer. The existing literature reviews of community-based screening initiatives have concluded that eliminating structural barriers such as location, distance, and inconvenient hours of operation is highly effective in increasing screening in high-income countries [6]. Some previous studies have found that older, uninsured, and African American women had higher odds of a repeat visit to the MSU compared with women who were younger, insured, and/or Caucasian [6]. Moreover, a study conducted in the United Kingdom found that men who were diagnosed with prostate cancer after screening at an MSU were more likely to be Black [7]. While mobile units are being adopted for cancer screening, research on their broader implementation and access-related outcomes is scarce. There is a lack of systematic reviews focused specifically on the design, implementation, and access-related patterns of mobile cancer screening programs. This review aims to synthesize the current evidence on the design and implementation considerations of mobile units for cancer screening services.

## 2. Methods

We conducted this systematic review following the Preferred Reporting Items for Systematic Reviews and Meta-Analyses (PRISMA) guidelines for its design, conduct, and reporting (See Table S2). The review aimed to synthesize empirical evidence on the reach, access-related outcomes, and implementation considerations of MSUs for cancer.

On 1 May 2025, we searched eight electronic databases: PubMed, Embase, Scopus, Web of Science, ProQuest, PsycINFO, IEEE Xplore, and ScienceDirect to identify relevant literature on mobile cancer screening services. Both free-text keywords and controlled vocabulary terms (e.g., MeSH) were used across databases. The review protocol was registered to the Open Science Framework [8]. Our review was informed by a PICO-style framework to clarify the population, intervention and outcomes of interest. However, because many eligible studies were descriptive or non-comparative, the review was not limited to studies with a formal fixed-site comparison group.

- Population: Adults eligible for cancer screening;
- Intervention/Exposure: Mobile unit-based cancer screening services;
- Comparison: Fixed-facility cancer screening services when available;
- Outcome: Screening uptake, reach, early detection, access-related outcomes, and implementation barriers.

### 2.1. Search Strategy

We developed our search strategy by combining keywords and controlled vocabulary terms (e.g., MeSH) related to mobile health units, cancer screening, and access to care. Boolean operators “AND” and “OR” were used to refine the search. An example query included terms such as (“mobile health clinic” OR “screening van” OR “mobile unit”) AND (“cancer test*” OR “cancer screen*”).

The search strategy was developed in consultation with a medical librarian to ensure completeness and reproducibility. The full search strategy for each database is provided in Appendix A. All retrieved references were exported to EndNote 20.1 (Clarivate, NJ, USA) for de-duplication and screening.

### 2.2. Inclusion and Exclusion Criteria

We included peer-reviewed articles that provided empirical evidence (qualitative, quantitative, or mixed methods) on the deployment of mobile units for cancer screening. Eligible publications met the following criteria: (1) focused on mobile units delivering cancer screening services; (2) targeted adults eligible for screening; (3) reported at least one relevant outcome (e.g., uptake, reach, access, early detection, or adherence); and (4) published in English in a peer-reviewed journal. We excluded articles that focused solely on fixed-site or hospital-based interventions, covered exclusively screening unrelated to cancer, or targeted pediatric populations. We did not include editorials, commentaries, letters, and papers without primary data.

### 2.3. Data Extraction, Study Selection, and Data Synthesis

Title and abstract screening, full-text review, and data extraction were conducted independently by multiple reviewers. At each stage, two authors independently reviewed the same records. Discrepancies were resolved through discussion and consensus, with a third author consulting when necessary. A standardized data extraction form was used to collect information on study characteristics, cancer type, screening modality, target population, outcomes, limitations, and implementation barriers. Data extraction and study selection were conducted independently by two reviewers. Inter-rater agreement was assessed through discussion and iterative comparison. Disagreements were resolved through consensus, with involvement of a third reviewer when necessary.

Due to the heterogeneity of study designs, populations, cancer types, and reported outcomes, a quantitative meta-analysis was not feasible. Therefore, a narrative synthesis approach was employed.

Studies were grouped and analyzed based on key characteristics, including cancer type (e.g., breast, lung, cervical, colorectal), screening modality, and study design. Descriptive findings were summarized to identify patterns in screening uptake, adherence, clinical outcomes, and population reach.

In addition, implementation-related findings were synthesized using a thematic approach as done in previous studies [9]. Reported barriers and challenges were extracted and iteratively categorized into three domains: (1) demand-side (client-level factors affecting uptake and follow-up), (2) supply-side (provider and operational factors related to program delivery), and (3) structural or policy-level factors (health system, policy, and infrastructure constraints).

This approach enabled the identification of recurring themes across studies while preserving contextual differences in study settings and populations.

### 2.4. Quality Assessment and Risk of Bias

Methodological quality was appraised using the Mixed Methods Appraisal Tool (MMAT) to characterize strengths and limitations across diverse study designs. Because the included literature was heterogeneous and frequently observational, MMAT ratings were used to inform interpretation rather than to imply uniform internal validity across studies. To aid interpretation, studies were categorized as having higher (A) or moderate (B) methodological rigor based on the extent to which MMAT criteria were met (n = 2 and n = 61, respectively); no studies were classified as having low methodological rigor. These classifications were used descriptively and do not eliminate important design-specific limitations, particularly for observational and non-comparative studies. One additional study was not appraised using the MMAT, as it primarily reported implementation-focused findings (e.g., lessons learned, operational challenges, and programmatic considerations) rather than empirical outcomes. It was included to provide contextual insight into real-world implementation [10].

To maintain consistency in quality-based inclusion criteria, this study was used exclusively for qualitative synthesis of implementation-related findings and was not included in quantitative comparisons or outcome-based interpretations. Because the included studies differed substantially in design, populations, screening modalities, and outcome definitions, the review was designed to identify recurring implementation patterns and access-related findings rather than to estimate pooled intervention effects or make strong causal claims about comparative effectiveness.

Quality appraisal was conducted independently by two reviewers. Inter-rater agreement was assessed through discussion and iterative comparison. Disagreements were resolved through consensus, with involvement of a third reviewer when necessary.

**Figure A1:**
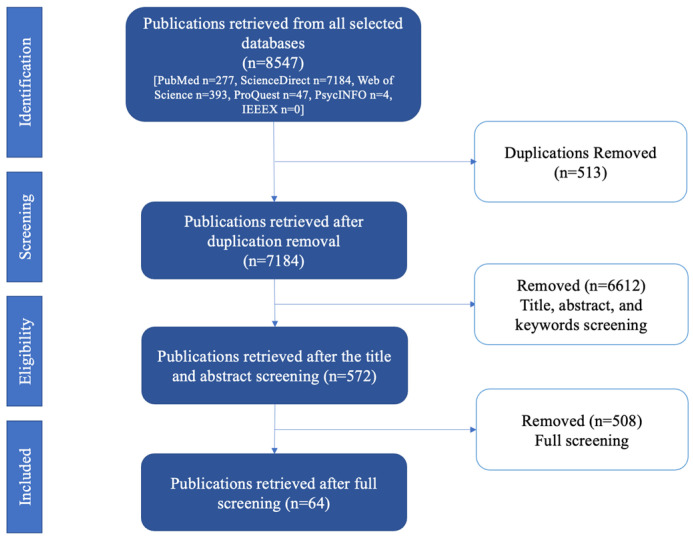
PRISMA Flowchart.

Backward snowballing was also performed by reviewing reference lists of included articles to identify additional relevant articles (see Appendix B, Figure A1).

## 3. Results

A total of 8547 articles were retrieved from the databases searched. After removing 7975 duplicates, we included 572 articles for screening. We screened the titles and abstracts of the articles and found 58 eligible articles. Six additional articles were identified using the reference snowballing method from reference lists of relevant articles, systematic reviews, and select cancer and public health journals. After a full article review, we deemed 64 articles eligible (58 from the initial search and six from snowballing) for data extraction, as shown in Supplementary Materials, Table S1.

### 3.1. Descriptive Results

Included articles were published between 1972 and 2025, with 20 being published before 2000 [11,12,13] and 19 since 2020 [14,15,16]. The studies took place in a total of 13 countries, with the U.S. containing the majority of articles (n = 30) [17,18,19], followed by the United Kingdom (n = 11) [10,20,21], and Brazil (n = 6) [22]. Geographical location was fairly evenly distributed, with 21 articles focusing on urban areas [23,24], 18 on rural areas [25,26], 17 on a mix of urban and rural areas [27,28], and eight not specifying the location of their target area [29,30].

Of the 64 articles included, 31 described quantitative descriptive studies, 27 detailed non-randomized quantitative studies, two were about randomized controlled trials, two described mixed-methods studies, one featured a qualitative study, and one did not fall into any of these categories as it was primarily implementation-science-focused [10].

### 3.2. Types of Mobile Screening Units Used in the Cancer Screening Promotion

Of the 64 articles included, 37 focused on breast cancer screening interventions [30,31,32], 11 on lung cancer [33,34], six on cervical cancer [12], three on skin cancer [35], three on colorectal cancer [36], two on prostate cancer [13], and two on multiple types of cancer [37]. Breast cancer: Of the exclusively breast-cancer-focused articles, all 37 involved mammography screening, while seven also included screening by clinical breast examination [38] and 13 included patient- and/or community-education components. Roughly half of these articles described interventions targeting women in their 40s and up [39], while a few included women as young as their 30s [26], only targeted older women [40], or did not specify the age of their target population [41]. A few also specified other target demographics such as race/ethnicity (e.g., American Indian, Latina), religion (e.g., Muslim), or socioeconomic status (e.g., low-income, uninsured, underinsured) [16,42,43,44].

Lung cancer: All 11 lung-cancer-related articles described interventions that used Computed Tomography (CT) screening [15,33], with seven also including other services such as spirometry, lung cancer risk calculation, patient education, and more [45,46]. Target populations varied considerably in age (with lower age limits ranging from 40 to 60), but most articles limited screening to current or former smokers who met age and pack-year eligibility requirements [23].

Cervical cancer: The six cervical-cancer-focused articles varied in their approach, with three describing screening via pap smear [47,48], two via visual inspection with acetic acid [49,50], and one not specifying the screening method [50].

Five also included patient education components and/or follow-up procedures such as colposcopy, cryotherapy, and loop electrosurgical excision procedure [50,51]. Target population characteristics were also varied for cervical cancer interventions, with lower age limits ranging from 16 [48] to 30 [48], with two articles not specifying an age eligibility criterion [51].

Skin cancer: Of the three skin-cancer-focused articles, one conducted teledermoscopy screening in agricultural workers 18 and older [35], while the other two did not specify their target populations (one of these involved screening by physical examination [52], while the other did not specify a screening method [52]).

Colorectal cancer: Among the three colorectal cancer articles, two focused specifically on individuals with Lynch syndrome or hereditary nonpolyposis colorectal carcinoma, to whom screening colonoscopies were provided [36], while the third provided general, abdominal, and rectal examinations (with rigid sigmoidoscopy) and various endoscopic procedures with a non-specific target population [29].

Prostate cancer and multiple cancers: Of the two prostate cancer articles, one provided digital rectal examination and measurement of prostate-specific antigen levels for men 45 and older with limited access to healthcare [53], while the other used transrectal ultrasonotomography for men over 55 [13]. Finally, both of the articles about several types of cancer used three or more screening methods on broad, non-specified target populations [37].

### 3.3. Role of the Units in Promoting Screening

Forty-eight articles examined either (a.) exclusively field-clinic-based (i.e., mobile) screening interventions (n = 45) [30] or (b.) screening interventions that were delivered in both field and traditional settings (without disaggregating results) (n = 3) [54]. Among these, 18 included inferential statistics [20], while 30 were limited to only descriptive findings [21]. The primary focus of the 18 inferential articles included (1) identifying predictors of screening attendance (i.e., uptake), screening reattendance, and/or follow-up attendance (n = 10) [6,55]; (2) assessing the effects of education and/or mobile screening interventions on screening-related behaviors (n = 4) [56]; (3) identifying predictors of screening-related clinical outcomes (e.g., abnormal screening results, being referred for additional services) (n = 2) [30]; and (4) identifying predictors of screening location preference (n = 2) [46]. A wide variety of associations were found between (re)attendance at screening/follow-up visits and hypothesized predictors such as demographics (e.g., age, race, insurance status, socioeconomic status) [38], distance of home from clinic site [54], and experience at previous screening appointments [57]. Educational interventions were primarily found to positively impact screening uptake [25] and screening-related knowledge [56]. Negative breast cancer screening clinical outcomes (e.g., being referred for additional services, receiving abnormal results) were found to be associated with uninsured status in both articles [19,30], and, in one study, with age under 50, Hispanic ethnicity, current smoking, and having a <50-year-old relative with cancer [30]. Of the two articles that examined predictors of screening location preference, one found that preference for a mobile (as opposed to hospital-based) lung cancer screening program was associated with being a current smoker and living in a socio-economically deprived area [46], while the other found that preference for mobile mammography or having no location preference (as opposed to preference for fixed facility screening) was associated with having had a prior mammogram [58].

Articles limited to descriptive statistics or qualitative findings (n = 30) generally focused on summarizing screening intervention attendance (i.e., uptake), clinical outcomes of the intervention, as well as client behaviors, perceptions, and experiences regarding screening. Commonly reported outcomes included the number of clients screened, type and number of abnormal findings, the cancer detection rate, recall rate (i.e., follow-up recommendation rate), follow-up attendance rate, and type and number of treatments performed [59]. In addition to these primary inferential or descriptive focuses, some articles also had secondary focuses, including summarizing (1) program cost or cost-effectiveness (n = 3) [17] and (2) lessons learned from program implementation (n = 1) [10]. Of the three articles examining the cost of their mobile screening programs, one found their program to be less costly than traditional facility-based screening [10], the second found their program to be financially viable (internal rate of return = 34.6%, profitability index = 2.2) [15], and the third calculated that program expenses would be <1% more than revenue generated [17]. The article that examined lessons learned primarily focused on barriers to program implementation discovered during the study [10], which are detailed in a later part of this review.

The remaining 16 articles examined field clinic versus traditional screening programs with the following foci: (1) comparing program-related factors (e.g., program uptake, clinical outcomes, accessibility) in mobile versus traditional screening programs (n = 11) [60]; (2) comparing general screening-related factors (e.g., attitudes, preferences, knowledge, behaviors) between mobile versus traditionally screened cohorts (n = 3) [61]; (3) comparing hospitalization duration and treatment costs between mobile-screened versus traditionally diagnosed cohorts (n = 1) [33]; and (4) identifying predictors of chosen screening type (i.e., mobile versus traditional) (n = 1) [27]. Of the 11 articles comparing program-related factors, a variety of results were found. In some cases, cancer detection or abnormal result rates were higher in facility-based screening programs [12], while in others, they were higher in mobile programs [28]. When compared, uptake was generally higher for mobile than facility-based screening [12]. Of the three articles examining general screening-related factors, all found lower screening rates or general adherence to screening guidelines in mobile screened populations compared to traditionally screened ones [61]. Screening-related knowledge was, in one case, higher among mobile-screened cohorts [61] and, in another, higher among traditionally screened individuals [48]. The article that examined hospitalization duration and treatment costs found asymptomatic individuals diagnosed with lung cancer through mobile screening to have had shorter hospital stays and lower treatment costs than symptomatic patients diagnosed in-hospital [33]. Finally, the article that examined predictors of chosen screening type found that women who were American Indian or Alaska Native (as opposed to another race/ethnicity), aged 50–64, lived in a rural area (as opposed to metropolitan or micropolitan areas), lived in the western U.S. (as opposed to other regions of the country), and lived in a community with average incomes under the national median had greater odds of having utilized mobile mammography at least once (compared to having only received facility-based mammograms) [27]. In summary, the evidence most consistently indicates more favorable patterns in access and screening uptake, while findings related to follow-up and clinical outcomes are more mixed.

### 3.4. Implementation Barriers

Twenty-one of the articles reviewed did not specify barriers to screening program implementation [34]. Thirty-one articles mentioned demand-side (i.e., client-level) barriers [29]; primarily highlighting factors that limit screening uptake, follow-up attendance, or the collection of complete and accurate data. The most common demand-side barriers were (1) population-specific barriers (e.g., age-related, cultural, regional), which limit screening acceptability and/or uptake (n = 16) [62]; (2) lack of client awareness of health-related factors (e.g., their own cancer risk, need for screening, need for follow-up) (n = 11) [63]; (3) general lack of follow-up compliance among clients (n = 8) [49]; (4) clients being unable to afford follow-up care (n = 4) [64]; and (5) client-side issues with this intervention specifically (e.g., concerns about the provided transportation services, privacy concerns when screening in mobile unit) (n = 3) [10].

Twenty-four articles reported supply-side (i.e., provider-level) barriers, which included factors concerning the implementation or delivery of the intervention [16]. The most common supply-side barriers were (1) cost of the intervention (e.g., start-up costs, equipment maintenance) (n = 10) [25]; (2) difficulty tracking follow-up care among clients who screened positive (n = 5) [59]; (3) issues with mobile screening units (e.g., cramped conditions, malfunctioning machinery) (n = 4) [37]; and (4) attracting sufficient amounts of clients (n = 3) [60].

Sixteen articles described structural or policy-level barriers [38], most of which were related to broader public health or healthcare systems. The most common structural barriers reported were (1) lack of physician support and/or referrals for screening among certain populations (e.g., mammograms for older women) (n = 5) [38]; (2) lack of physician training programs related to the type of screening in question or general need for specifically trained physicians to participate in intervention delivery (n = 3) [26]; (3) lack of insurance coverage or reimbursement for screening (n = 3) [27]; (4) national government not prioritizing screening (e.g., lack of national guidelines around screening, screening is not a policy priority) (n = 3) [60]; (5) difficulty with identifying clients in need of screening (e.g., due to lack of national patient registry) (n = 3) [60]; and (6) geographic, distance, and/or transportation barriers inhibiting follow-up attendance (e.g., lack of local healthcare infrastructure) (n = 3) [39]. In summary, findings suggest that barriers clustered around follow-up coordination, infrastructure, and policy/reimbursement constraints across settings.

### 3.5. Limitations of the Units Based on the Included Articles’ Findings

Twelve articles did not discuss study limitations [13]. Among the 52 that did, 39 mentioned methodological limitations, such as factors related to study design or data analytics [51]. The most common methodological limitations were (1) not collecting information on certain potentially relevant variables (e.g., body mass index, race/ethnicity) or not considering the effects of these variables in analyses (n = 23) [24]; (2) limited generalizability of results (due to methodological reasons) (n = 10) [65]; (3) potential systematic over- or under-estimation of one or more variables (due to methodological reasons, such as methods used to calculate certain measures) (n = 7) [66]; (4) reliance on self-report data (n = 5) [67]; and (5) short study or follow-up period (n = 5) [68].

Only 11 articles discussed implementation-related limitations [43] and generally focused on unique, study-specific factors. However, two articles noted the possibility that screening staff may have missed some cancer cases, resulting in false-negative screening results [35]. Other examples of implementation limitations included the inexperience of clinical staff delivering the intervention due to a short training period [52], inability to control parking for the mobile unit [39], and inability to verify whether screening invitations had been received [21]. Other study limitations that were noted, but for which reasons were not always specified (e.g., methodological versus implementation-related cause), included the sample population not being completely representative of the target population (n = 22) [23], missing data (n = 15) [31], and a small sample size (n = 10) [28].

## 4. Discussion

Our review study synthesized evidence from 64 eligible articles to examine the use of mobile units in cancer screening promotion and delivery across multiple cancer types, geographic contexts, and healthcare systems. Collectively, our findings indicate that these units play a distinct role in cancer prevention efforts, particularly in settings characterized by structural barriers to care. Because most included studies were observational, descriptive, or otherwise non-randomized, the findings should be interpreted as evidence of recurring implementation patterns and access-related associations rather than definitive evidence of comparative effectiveness.

### 4.1. Mobile Screening and Access to Care

Several studies reported high uptake in mobile screening programs, but interpretation is limited by differences in populations served, study design, and the likelihood of confounding [12,27,28,60]. This pattern is consistent with the possibility that decentralizing preventive services can help reduce geographic and logistical barriers to care, particularly among populations with limited access to fixed healthcare facilities [69,70]. Compared to earlier program-specific reviews, our study extends existing knowledge by suggesting that these access-related benefits are observed across multiple cancer types, including breast, lung, cervical, and colorectal cancers [30,31,32,33,34,36], and across diverse international contexts. However, several comparative studies included in our review also reported differences in adherence and clinical outcomes between mobile and facility-based programs [12,61]. These disparities likely reflect systematic differences in the populations served, as mobile screening cohorts frequently exhibited lower baseline screening adherence and greater socioeconomic disadvantage [26], aligning with equity-focused frameworks, cautioning against direct performance comparisons between interventions serving structurally different populations [71,72]. Importantly, differences in adherence and clinical outcomes between mobile and facility-based screening programs should not be interpreted solely as indicators of intervention effectiveness. Many studies included in this review involved populations with differing baseline characteristics, including socioeconomic status, insurance coverage, and prior engagement with healthcare systems. As a result, lower adherence observed in mobile screening cohorts may reflect underlying structural barriers rather than limitations of the intervention itself. These findings highlight the need to interpret comparative outcomes within the broader context of population differences and access inequities. In addition to these contextual differences, several methodological considerations further shape the interpretation of these findings. Confounding is likely present across many included studies, as key factors such as socioeconomic status, insurance coverage, comorbidities, and prior healthcare engagement may influence both participation in mobile screening and subsequent outcomes. Moreover, selection bias is inherent to many mobile screening programs, which are often intentionally deployed to reach populations with historically lower screening uptake or limited access to care. In addition, these cross-cutting patterns should be interpreted in light of the evidence base’s strong concentration in breast cancer screening studies. As a result, participants in mobile screening initiatives may systematically differ from those accessing facility-based services.

Finally, it is critical to distinguish between intervention performance and the structural conditions under which these programs operate. Mobile screening units frequently serve populations affected by structural inequities, including geographic isolation, transportation barriers, financial constraints, and healthcare mistrust. These structural factors can directly influence adherence, follow-up, and clinical outcomes, and may partially explain differences observed across studies. Therefore, outcome differences should be interpreted within this broader structural context rather than attributed solely to the effectiveness of the mobile screening intervention.

### 4.2. Engagement, Outreach, and Contextual Effectiveness

Another key result of our review is the importance of community context in shaping the outcomes associated with mobile cancer screening programs. Studies reporting high uptake (based on their outcomes) frequently described deployment at trusted or high-traffic community locations and integration with outreach or education efforts [26,38]. Prior public health research and theories have also highlighted the role of trust, community involvement, and cultural relevance in the success of preventive health behavior initiatives [73].

While previous literature has acknowledged the importance of education and outreach in screening promotion [74,75,76], our review expands on that by suggesting that such strategies may contribute to more favorable uptake patterns in mobile screening contexts, where the intervention itself may represent the first or only point of contact with the healthcare system for some populations.

### 4.3. Design, Implementation, and Feasibility Considerations

We also identified recurring implementation-level factors influencing the feasibility and acceptability of the MSUs, including physical space constraints, equipment reliability, staffing, and workflow efficiency [37,40,52]. Our results align with implementation science literature emphasizing that program performance and implementation outcomes are shaped not only by clinical content, but also by delivery context and operational design [77]. By synthesizing implementation-related barriers across studies, we extend prior work that has typically reported these challenges in isolation. Importantly, the presence of such barriers does not diminish the value of mobile screening programs; rather, it highlights the need for deliberate attention to design and implementation processes to ensure sustainability [77].

### 4.4. Downstream Care and Referral Pathways

One of the persistent challenges that our study captured was the ability to ensure follow-up care after mobile screening, particularly for individuals with abnormal results [68]. This issue was explained by difficulties related to tracking patients, coordinating referrals, and ensuring diagnostic resolution. This result is consistent with earlier research identifying follow-up as a key limitation of community-based and mobile screening programs [78].

However, several studies reported more favorable outcomes in mobile screening programs that were supported by clear referral pathways and linkage to fixed healthcare facilities [10,33]. These findings emphasize that screening program outcomes should be evaluated not only on initial uptake, but also on downstream continuity of care.

### 4.5. Practical Implications

**Figure 1:**
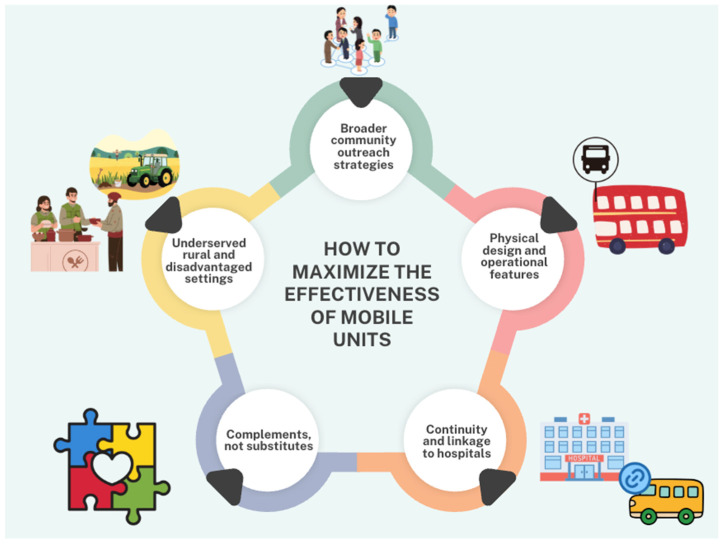
Emergent lessons for the design and deployment of mobile screening units.

This review identified recurring patterns across studies that provide insight into how mobile screening units are deployed, the contexts in which they may be most useful, and considerations for their design and implementation. Figure 1 summarizes these emergent lessons.

Mobile units were most commonly deployed in underserved urban areas. In these settings, their access-expansion potential may be greatest, particularly where geographic distance, transportation barriers, or limited local screening infrastructure restrict access to facility-based care. Several studies reported higher screening uptake in these settings; however, these findings should be interpreted cautiously, given the limited number of direct comparative studies and differences in populations served and study design, particularly in studies examining rural settings.

Importantly, mobile units may function differently across urban and rural contexts. In urban settings, they may improve convenience, visibility, and community trust, or help address gaps in access among underserved populations. In contrast, in rural settings, they may serve as a partial substitute for limited or absent screening infrastructure. More favorable outcomes were often observed among populations with historically lower adherence to care services, including uninsured individuals, racial and ethnic minority groups, and residents of socioeconomically deprived areas; however, these patterns may reflect targeted deployment and underlying population characteristics rather than differences in intervention effectiveness. Taken together, these findings suggest that mobile units may be particularly useful as an access-expansion strategy within existing health systems, rather than as a replacement for facility-based screening.

In addition, units that were stationed at familiar, high-traffic, or trusted community locations (e.g., community centers, workplaces, faith-based sites, agricultural settings, or community events) were often associated with stronger engagement and higher attendance. Studies that paired mobile units with community education, patient navigation, or culturally tailored outreach were associated with more favorable uptake and knowledge outcomes. compared to screening-only approaches. These findings suggest that mobile units may be more useful when embedded within broader community-engaged screening strategies, rather than operating as standalone clinical services.

It is also noteworthy that some of the included studies emphasized the role of physical design and operational features of mobile units in influencing patients’ experiences, provider efficiency, and overall feasibility. For example, adequate privacy, sufficient interior space, reliable equipment, and efficient patient flow were repeatedly cited as facilitators of acceptability and sustainability. On the other hand, cramped conditions, equipment malfunctions, and privacy concerns in the mobile setting were identified as barriers to both client participation and staff performance. These results highlight the importance of designing mobile units not only to meet technical screening requirements but also to support dignity, confidentiality, and provider workflow, particularly for sensitive screenings.

For screening, while many units were successful at initiating screening, several studies reported challenges with follow-up, especially in tracking patients with abnormal results. More favorable outcomes were often observed in programs with clear referral pathways, structured follow-up processes, and linkage to fixed healthcare facilities. These findings suggest that outcomes associated with mobile units may be more favorable when follow-up systems are integrated and when mobile units are positioned as entry points into a broader continuum of care rather than as isolated screening encounters.

Finally, in some studies, differences in clinical outcomes and adherence patterns were associated with systematic differences across populations (e.g., lower baseline screening adherence, higher structural barriers to care, etc.). This observation suggests that mobile units serve a distinct and complementary role within cancer screening systems. Rather than replacing facility-based programs, mobile units appear most beneficial when strategically deployed to close access gaps, reach populations unlikely to attend traditional screening, and extend services into high-need settings.

### 4.6. Limitations

Our review has several limitations that are worth acknowledging. First, the review included a relatively large number of heterogeneous studies spanning multiple cancer types, study designs, and geographic regions, which limited our ability to compare findings across studies directly or to draw causal inferences. In addition, outcome reporting was inconsistent across studies. While screening uptake was frequently reported, fewer articles systematically documented follow-up, time to diagnosis, or treatment initiation after abnormal screening results. The consistent absence of follow-up and treatment initiation outcomes reflects not only reporting gaps but a structural limitation of stand-alone mobile screening models. As a result, the review may overrepresent evidence related to screening initiation relative to continuity of care and long-term outcomes. The evidence base was also dominated by breast cancer screening studies, which may limit the generalizability of findings to other cancer types with different screening processes, infrastructure requirements, and follow-up pathways. Our review is also subject to reporting bias. Additionally, one implementation-focused study that was not appraised using the MMAT was included to provide contextual insights; however, its contribution was limited to qualitative synthesis and did not influence comparative or outcome-based conclusions.

Moreover, some articles did not explicitly report implementation barriers or limitations, potentially leading to an underestimation of the real-world challenges associated with mobile screening deployment. Across the included studies, the predominance of observational and non-comparative designs introduces important limitations in causal interpretation. Many reported associations between mobile screening and outcomes such as uptake or adherence are likely influenced by confounding factors, including socioeconomic status, geographic access, and healthcare infrastructure. Selection bias is also a key consideration, as mobile screening programs are often deployed in underserved communities with historically lower screening participation. Consequently, observed differences between mobile and facility-based programs may reflect differences in population characteristics rather than true intervention effects. Future research should incorporate more rigorous comparative designs to disentangle these factors and better isolate the impact of mobile screening interventions.

## 5. Conclusions

This review contributes to the literature by synthesizing a heterogeneous body of evidence spanning multiple cancer types, screening modalities, and healthcare systems. Unlike prior reviews that focus on single cancers, specific screening programs, or isolated delivery models, this work provides a cross-cutting assessment of mobile screening units (MSUs) as access-oriented interventions operating across diverse contexts. By examining how MSUs are deployed to address structural barriers, such as geographic access, insurance coverage, and healthcare mistrust, this review highlights their potential role within broader cancer prevention and early detection strategies.

At the same time, important gaps remain in the existing evidence base. Many studies rely on descriptive or observational designs, with limited use of inferential or comparative approaches that would allow stronger causal inferences about comparative performance. Reporting of follow-up outcomes, including diagnostic resolution, treatment initiation, and longitudinal adherence, is inconsistent, and follow-up periods are often short. These limitations constrain the ability to assess the sustained contribution of MSUs to cancer outcomes and health access, underscoring the need for more rigorous study designs, standardized outcome measures, and longer-term evaluations in future research.

## Data Availability

No new data were created or analyzed in this study. Data sharing is not applicable to this article.

## Supplementary Materials

The following supporting information can be downloaded at: https://www.mdpi.com/article/10.3390/ijerph23040465/s1, Table S1: Results Table, Table S2: PRISMA 2020 Checklist.

## Appendix A. Search Expressions

**Mobile van/clinics/screening keywords:**
  “Mobile Health Unit” OR “Mobile Clinic” OR “Mobile Health” OR “Mobile Van” OR “Mobile Health Clinic” OR “Mobile Medical Clinic” OR “Mobile Health Van” OR “Mobile Medical Unit” OR “Mobile Medical Van” OR “Mobile Screening Unit” OR “Mobile Imaging Unit” OR “Mobile CT Unit” OR “Mobile CT Scanner” OR “Mobile Diagnostic Unit” OR “Mobile Radiology Unit” OR “Pop-Up Clinic” OR “Traveling Clinic” OR “Traveling Health Unit” OR “Health Screening Van” OR “Healthcare Delivery Van” OR “Mobile Outreach Unit” OR “Mobile Healthcare Service” OR “Mobile Preventive Health Service” OR “Community Screening Van” OR “Community-Based Screening Unit” OR “Mobile Health Intervention” OR “Mobile Screening” OR “Rural Health Van” OR “Outreach Clinic” OR “Rural Mobile Clinic” OR “Urban Mobile Clinic” OR “Field Clinic”

**General cancer screening keywords:**
  “cancer screen” OR “cancer detect” OR “screening for cancer” OR “oncology screen” OR “oncological screen” OR “low dose ct” or “low-dose ct” or LDCT or “low-dose computed tomography” OR “MRI screening” OR “MRI” OR mammography OR “CT screen” OR colonoscopy OR “pap smear” OR “HPV test” OR “genetic cancer test” OR “FIT test” OR “PSA test” OR “cancer biomaker” OR “cancer risk”
