# Supplementary material for: Mobile Cancer Screening Programs: A Systematic Review of Implementation Challenges and Population Access
Source: Int J Environ Res Public Health. 2026 Apr 4;23(4):465. doi: 10.3390/ijerph23040465 (PMC13116370; doi:10.3390/ijerph23040465)
Supplement: Supplementary file 1 [file ijerph-23-00465-s001.zip › ijerph-4211489-supplementary Table S1.pdf]

Supplementary file

Table S1. Results Table.

Title	year	Journal	Setting (field clinic vs traditional, fully field)	Country	Target area of the study and Is it a rural or urban setting or both, is it low-income or high-income area, etc	what is the target population?	Type of cancer screened	Type of screeningperformed (mamo, LDCT, LEEP, etc)	Is it single screen only or different screening services?	intervention characteristics (description of the mobile clinic including staffing, drugs, equipment, number of visits to the target area etc.)	Characteristics of the providers helping (profession, level of training, age, etc) in both types of clinics	Design of the mobile unit if mentioned	Screening process (is it done there or it just covers referral process, etc)	Facilitators of implementation of the outreach intervention	Barriers of implementation of the outreach intervention	Health outcomes measured (knowledge, access, utilization, mortality, access, coverage, etc)	Quantification of the previously mentioned variables (FINDINGS). PS: if there is a comparison please mention both mobile and traditional clinics' findings	Limitations of the study
a mobile mass-screening unit for prostat	1984	PROSTATE	Field	Japan	Keihoku-cho town, Kyoto Prefecture (Rural	Males, 55 years and older	Prostate cancer	Transrectal ultrasonotomography, digital palpation,	Multiple screening ser	Not specified	Not specified	Mobile unit (bus) called "Dolphin" Floor	Conducted in mobile unit		Not specified	Cases of BPH Cases of prosta	Detected 463 cases (33.2%) of benign prosta	Not specified

ic diseas e					) and Sugau ra hamle t in Nishia sai town, Shiga Prefec ture (Rural)			measur ement of residual urine, x-ray procedu res, prostati c needle biopsy	vic es			area is 8m^2 Oper ation conso le in the cente r with two chair-type scann ers are fixed on either side Wast e buck ets acces sible from outsi de for dispo sal and are adjac ent to electr icity cable s for conn ectio n to			tic cancer	tic hypert rophy (BPH) Detect ed 8 cases (0.6%) of prosta tic cancer	

												outside power source No undressing area inside						
factors affecting womens response to an invitation to attend for a 2nd breast-cancer screening examination	1991	BRITISH JOURNAL OF GENERAL PRACTICE	Field	United Kingdom	Aylesbury (urban) Aylesbury Vale (primarily rural)	Women, 45-64 years who were registered at one of three general practices in Aylesbury	Breast cancer	Mammography	Single screening service	Women invited to screening by doctor Women screened on Tuesdays and Thursdays were included in the study	Not specified	Not specified	Not specified	Not specified	Not specified	Views about breast cancer screening experience Beliefs about breast cancer and breast cancer screening	48 women (6.6%) found last screening embarassing, 46 women (6.4%) found last experience distressing, 188 women (26.1%) found last screening	Not specified

																	painful, 29 women (4.0%) found staff not helpful, 21 (3.3%) found staff unsupportive<br><br>681 women (94.8%) found test reassuring 694 (96.0%) felt that previous attendance had been worthwhile	

addressing cervical cancer disparities in texas: expansion of a community-based prevention initiative for medically underserved populations	2023	prev med rep	Field and clinic	United States	Medically underserved areas of Texas (rural)	"Eligible women"	Cervical cancer	Colposcopy, cervical biopsy, LEEEP	Multiple screening services	Each site was staffed with a patient navigator from local community Women with abnormal screening tests were contacted by medical providers Patient navigators assisted with scheduling follow up appointments and sent reminders	Patient navigators received standardized training and mentoring led by the MD Anderson team	Not specified	Conducted in mobile unit/participating clinic	MD Anderson, UTHealth	Limited number of trained health care personnel (provider/system level) Insufficient funding to expand screening and prevention services (provider/system level) Difficulty navigating the health care system (patient level)	Patient education Patient screening	Patient navigators educated 75,842 women Screened 44,781 women 658 women diagnosed with high-grade dysplasia 33 women diagnosed with cancer and referred to oncologic care	Used the first 6 months of data collection as baseline for comparison No control group Difficult to assess impact of individual intervention components because of multicomponent nature Data were collected in aggregate -- no individual-level data available for analysis

															Inability to miss work (patient level)<br>Limited transportation and access to childcare (patient level)<br>Fear of deportation (patient level)			

socioeconomic deprivation, travel distance, location of service, and uptake of breast cancer screening in north derbyshire, uk	2006	Journal of Epidemiology and Community Health	Field and clinic	United Kingdom	North Debyshire (rural)	Women, 50-64, resident in North Derbyshire	Breast cancer	Mammography	Single screening service	Intervention provided at 13 locations of "urban depreivation"<br>1 site at a Chesterfield-based hospital<br>12 sites at mobile breast cancer screening units<br>9 sites at health facilities, 3 sites at council tax office, swimming pool, leisure center parking lot<br>Screening once every 3 years	Not specified	Not specified	Women screened from April 1998 to March 2001 (3-year period) once per year	Breast cancer screening uptake	Uptake 73% in the most socioeconomically deprived category, 82% in the least deprived category<br>Association between socioeconomic deprivation and odds of BCS attendance (OR=0.64, 95% CI 0.59-0.70, p<0.0001)<br>Association betwe	Misclassification at individual level 2/2 attributing small area level socioeconomic deprivation score to all women within the care<br>Errors calculating distance from screening site.<br>Could not differentiate between incident and prevalt screens<br>Did not consider effect of age on uptake<br>No information on the use of mammography services

															en living far (≥8km away from location) and BCS attendance (OR = 0.89, 95% CI 0.81-0.99) compared to women living 2km from screening location<br>Type of screening facility had no effect on attendance (OR = 1, 95% CI 0.94-	

																	1.07) for attendance at fixed site vs mobile unit Urban rural status vs attendance (OR = 1.0, 95% CI 0.91-1.09)	
breast cancer incidence in mobile screening vs. in-hospital screening programmes based on 6313607	2024	Journal of Global Health	Field and clinic	Taiwan	Rural and urban	Women, 46-69	Breast cancer	Mammography	Single screening service	Not specified	Certified radiologists who have completed an educational course (with a grade of 'B' or higher) on	Mobile units equipped with either computed radiography or digital radiography	Not specified	Radiologists (only individuals allowed to interpret findings of images)	Not specified	Recall rate: proportion of screening examinations assessed as BI-RADS Cancer Detection	Prevalence Screen Hospital CR recall rate = 3.3 Hospital CR PPV = 22.1 Hospital CR CDR = 7.24 Hospital DR	Because of retrospective design, lacked prospective randomization Potential introduction of selection bias (time bias) Short follow-up period

mammograms in 2387756 women in taiwan											mammogram interpretation or quality control, earning 10 training credits annually, reviewing certification every 2 years					Rate (CDR): number of screenings detecting breast cancer per 1000 performed Positive predictive value	recall rate = 5.0 Hospital DR PPV = 21.2 Hospital DR CDR = 10.60 Mobile CR recall rate = 3.1 Mobile CR PPV = 19.9 Mobile CR CDR = 4.38 Mobile DR recall rate = 4.3 Mobile DR PPV = 13.8 Mobile DR CDR 5.91<br><br>For subsequent screen (in the	

																same order as above 2.2, 19.9, 4.38 3.2, 17.8, 5.71 2.2, 13.2, 2.97 2.9, 12.8, 3.74<br><br>In-hospital screening CDR (10.6 per 1000) Mobile screening CDR (4.75%)	
community education programs to promote mamm	1998	Preventive Medicine	Field	United States	New York State (rural)	Women, 35+	Breast cancer	Clinical breast examination, mammography	Multiple screening ser	Films processed on board van Results interpreted within	Nurse and mammography technologist on the van,	Not specified	Conducted in mobile unit	Not specified	Not specified	Evaluation of intervention impact Impact on	62% overall response rate<br><br>Impact on mam	Study design did not provide all assurance of internal validitiy from formal trial (no

ography participation in rural new york state									vices	48 hours by board-certified radiologists and reported to PCP	board-certified radiologists for interpretation of screening					mammography<br>Impact on CBE<br>Impact on BSE	mography<br>- more women from the program area reported receiving their most recent mammogram in the past 2 years (82% vs 72%, p<0.01)<br>- more women from the program area reported receiving their most recent mammogram in the	community trial)<br>Lack of formal pretest; no suggestion of differences in in breast cancer screening behaviors at baseline<br>Relatively brief followup period of 6 months

																	past year (64% vs 60%, p = 0.03)<br>- more women from the pgram area indicated that they receive mammography regularly (55% vs 51%, p = 0.04)<br>- more women in Program area indicated that they had received mammogram from	

																	mobile van (34% vs 10%, p<0.01)<br><br>Impact on CBE<br>- no difference in CBE behavior (75% vs 78%, p = 0.10)<br>- most recent mammography completed by nurse or nonphysician (29% vs 21%, p = 0.01)<br>- higher use of mobile van?<br>- CBE	

																	mattering to friends in Program vs Comparison areas (75% vs 62%, $p < 0.01$)<br><br>Impact on BSE<br>- no significant program effect of regular BSE performance (56% vs 55%, $p = 0.90$)<br>- significant program effect for knowledge of recom	

																	mended BSE frequency (72% vs 67%, p = 0.02)<br>- significant program effect for confidence in ability to do BSE (74% vs 65%, p = 0.02)<br>- significant program effect for reporting being taught BSE within the past year (41%	

																	vs 31%, p = 0.02)	
the chiraiya project: a retrospective analysis of breast cancer detection gaps addressed via mobile mammography in jammu province, india	2024	BMC Public Health	Field	India	Jammu Province, India (rural)	Women, 40+ in Jammu Province, India	Breast cancer	Mammography	Single screening service	Administration of structured questionnaire, mammographic examinations, post-mammographic examination questionnaire medical officer, nurse, trained technologists, radiologists, driver, "helper"	Technologists trained under strict quality control measures	Equipped with state-of-the-art mammographic equipment (high-resolution digital imaging systems)	Conducted in mobile unit	Screening camps organized in collaboration with various NGOs, army camps, village panchayats, and urban cooperatives	Significant loss to follow-up 2/2 education, awareness, affordability	BIRADS classifications Follow-up rates Regional variations	BIRADS classifications - 32.96% in 0 category - many patients need additional imaging - 11.1% in I category - had negative findings - 48.77% in II category - 4.45% in III catego	Exclusion of data from 2020 to 2021 2/2 COVID-19 introduced bias and affected generalizability Reliance on mammography as sole screening modality led to overlooked cases of breast cancer not detectable by this method

																	ry<br>- 2.13% in IV category<br>- 0.60% in V category<br><br>Follow-up rates<br>- Overall, 404/604 (66.89%) were followed up (200 (33.11%) were LTF)<br>- For BIRADS 0, 320/496 were followed up<br>- For BIRADS V, 100% follow-up	

																	rate<br><br>Regional variations<br>- rural areas had higher screening uptake<br>- urban areas had higher follow-up rates<br>- urban areas have greater awareness of BC	
mobile lung screening: should we all get on	2020	Annals of Thoracic Surgery	Field	United States	Tennessee (rural)	Men and women, 50-86 (mean 62) Mean pack-years =	Lung cancer	CT	Single screening ser	CT scanner 104 sites visited	Not specified	Winnebago speciality vehicle com	Conducted in mobile unit		Slow implementation 2/2 skepticism with	Lung-RADS category Cancer characteristi	The bus traveled to 104 sites and screen	Employers punishing smokers with higher health insurance premiums Tennessee's

the bus?						41 (1-110)			vice			mercial shell Modified Siemens 16-slice Somatom Scope CT scanner		new screening tools Smokers' hesitation to screen at all	cs identified Nonpulmonary findings	ed 548 patients over 10 months in 2018<br><br>Lung-RADS category<br>- 1 in category 0<br>- 303 (55%) in category 1<br>- 193 (35%) in category 2<br>- 33 (6%) in category 3<br>- 18 (3%) in category 4<br>- 54/232 (23%) of patients with nodul	rural geography Cost to purchase and run a mobile screening program (financial sustainability)

																es >2mm required further testing<br>- Of the 54, 49 (91%) required additional CT or PET scans<br><br>Lung cancers identified<br>- 2 cancers were stage 1A<br>- 1 cancer was stage 1A2<br>- 1 cancer was stage 1B<br>- 1 cancer was stage	

																	3A<br><br>Nonpulmonary findings were found in 152 (28%) of persons screened	
mobile colonoscopic surveillance provides quality care for hereditary nonpolyposis colorectal carcinoma families in south africa	2007	Colorectal Disease	Field and clinic	South Africa	Endoscopic unit in Cape Town (urban)<br>Mobile endoscopic service at hospitals on West Coast of SA (rural)	Asymptomatic subjects with known disease-causing mismatch repair gene mutation	Hereditary non polyposis colorectal carcinoma	Colonoscopy	Single screening service	Colonoscopy performed under conscious sedation	Gastroenterologist, colorectal surgeon, endoscopy trained nurses, coordinator, histopathology technician, equipment management staff	Not specified	Conducted in mobile unit/participating clinic	Participants recruited from Colorectal Surgery Unit and Division of Human Genetics from University of Cape Town	Not specified	Number and interval of colonoscopies<br>Whether colonoscopy was complete or not<br>Results of repeat colonoscopy<br>Histopathological assessment of	259 colonoscopies performed in 91 subjects<br>171 performed in mobile clinic in 60 subjects<br>88 performed in endoscopic units	Not specified

																colonoscopic biopsies<br>Results of any surgical intervention<br>Colorectal cancer deaths	in 30 subjects<br><br>249 colonoscopies were complete (96%)<br>8/91 subjects with initial screening colonoscopy had positive screening (8.8%)<br>- 4/60 of positive results obtained on West Coast<br>- 4/31 in endoscopic units<br>Of remaining	

																	negative colonoscopy,<br>- 62 entered surveillance colonoscopy program<br>- 41 on West Coast<br>- 21 in Cape Town<br>- 186 total surveillance colonoscopies performed<br><br>13/62 subjects in surveillance had positive result (21%) after media	

																	n of 3.1 colonoscopies<br>- 9/116 of positive results obtained on West Coast<br>- 4/52 obtained in Cape Town<br><br>3 deaths occurred during the study period	
mobile mammography: assessment of self-referral in reaching medically unders	2007	Journal of the National Medical Association	Field	United States	Cook's county (urban/suburban)	Women, 40+ with no current breast cancer symptoms	Breast cancer	Mammography	Single screening service	Free screening mammography Patients and providers mailed screening reports within 14 days	Not specified	Not specified	Conducted in mobile unit	Films interpreted by John Stroger Hospital of Cook County	Not specified	Mobile Mammography Community Site Visits Patterns of Care for Diagn	321 sites visited between 1999 and 2002<br>- 163 community health centers	No data on siocioeconomic status Recruitment survey response rate of 55.5% (results do not reflect those of the most medically

erved women										of procedure Women prospectively followed for 12-24 months						ostic Follow-Up Safety-Net Healthcare Utilization Patterns	(50.8%)<br>- 80 community-based organizations (24.9%)<br>- 28 government agencies (8.7%)<br>- 26 public housing developments (8.1%)<br>- 19 community health fairs (5.9%)<br>- 5 private companies (1.6%)<br><br>636 women with abnormal	underserved)

																	screening mammograms<br>- 20% obtained follow-up testing<br>- 29.5% consulted w/ private physician<br>- 54.1^ sought care at a hospital outside of safety-net health system<br>- Reasons for follow-up at non-safety-net sites<br>- adequ	

																	ate insurance coverage/concerns about health plan regulations (16.4%)<br>- proximity to another facility (20.5%)<br>- established provider at different facility (46.6%)<br>- logistical and bureaucratic barriers in obtain	

																	ing county hospital appointment (12.3%)<br>- preference for care outside safety-net system (19.9%)<br><br>32.7% of women accessed health care services within safety-net prior to mammogram <1%	

																	had received mammogram<br>- After mammograph, 37.9% subsequently accessed medical care at safety-net<br>-	
opportunistic screening for skin cancer using a mobile unit in brazil	2011	BMC Dermatology	Field and clinic	Brazil	Amazonas region of Brazil (rural and urban)	Men and women, 40+	Skin cancer	Physical examination for skin cancer screening	Multiple screening services	Mobile unit team able to perform 40 physical examinations of the skin per day suspicious lesions subjected to histopathological evaluati	Clinical or surgical oncologist, nurse, 3 nursing assistants, driver	Not specified	Conducted in mobile unit	Barretos Cancer Hospital	Reduced time to perform screening during regular physical examination lack of specific training program for dermatologists to recognize lesions that are potentially neoplastic		17857 consultations carried out 3005 biopsies/surgeries performed 2012 (11.2%) cases of skin cancer diagnosed 82% with	Lack of medical oncologists and dermatologists in visited cities Limited experience of nurses 2/2 short training time

										on, local resection with margins, or local resection with skin flaps or skin graft Patients with large lesions were scheduled to be treated at BCH						skin cancer had resection performed in the MU 74.6% had one suspicious lesion that was surgically removed Biopsy performed in 230 (11.4%) patients 132 (6.6%) patients had no surgery in MU and were referred to BCH Of	

																	3005 suspicious lesions analyzed, 67% showed evidence of cancer (predominantly basal cell carcinoma (81.6%) 93.9% stage 0 or I 5.5% stage II 0.6% stage III	
estimating lifetime risk for breast cancer as a screening tool for	2022	Journal of Cancer Policy	Field	United States	New York City (urban)	Women, 40+	Breast cancer	Mammography	Single screening service	Not specified	Not specified	Not specified	Conducted in mobile unit/participating clinic	Mount Sinai	Not specified	Average ten-year risk score Average lifetime risk score	2195 total screened 2.79% of women reported havin	Self-reported measures vulnerable to social desirability and recall biases Translation services could have

identifying those who would benefit from additional services among women utilizing mobile mammography																Recommendation for additional services	g breast problems at time of screening Average TC ten-year risk score 2.76% ± 2.01% Average TC lifetime risk score 7.30% ± 4.80% 444 (20.23%) patients could be referred for additional services Odds of being recommend	led to potential miscommunications NYC sample may not have represented racial and ethnic population

																	ed for additional services by the TC model (not significantly different from those living in manhattant - Brooklyn OR = 1.18 (0.82-1.70) - Bronx OR = 0.97 (0.71-1.32) - Queens OR = 1.15 (0.86-1.53) - Staten Island OR = 1.17 (0.72-1.90)	

																	Odds of being recommended for additional services by the TC model were significantly greater among those eligible for CSP when compared to those ineligible, OR = 1.31 (1.03-1.66)<br>- After adjustment, OR = 1.37 (1.03-1.81)	

herault breast screening programme: results after 30 months of a mobile french schedule	1995	European Journal of Cancer Prevention	Field	France	Montpellier (urban)	Women, 40-70	Breast cancer	Mammography	Single screening service	Screening every 2 years Mammographies tarnsmitted daily from mobile van to IMIM For all cancers detected, referral to treatment provided	Radiologists (trainedspecifically in examining mammographic films)	Not specifed	Conducted in mobile unit	Institut Montpellierain d'Imageria Mediciale (IMIM)	Not specified	Participation rate Quality of screening and diagnosis strategy	Participation rate - 26026 women attended mammography in two mobile vans between July 1990 and December 1992 (48% participation rate)<br><br>Quality of screening and diagnosis strategy - 1826 women (7%) recalled for furthe	Participation rate needs to be improved to reach European standards

																	r examination - Of the 383 patients referred for biopsy or survery, 135 found to have histologically confirmed cancers	
breast cancer detection rates by screening mammography in elderly women	1997	Breast Journal	Field and clinic	United States	Outpatient clinic at Russell Ambulatory Center (urban) Mobile van (rural)	Women, 50+	Breast cancer	Mammography	Single screening service	Screening conducted in mobile van and outpatient clinic Mammographic results interpreted by radiologists	Radiologists are board-certified	Van equipped with Siemens Mammomat 2 Outpatient clinic equipped with CGR 500% and Xerox	Conducted in mobile unit	University of Alabama at Birmingham (UAB) for the mobile van Russell Ambulatory Center for the UAB outpatient clinic		Cancer-detection rate	Cancer-detection rate among women aged 65+ is twice as high at the van, and three times has	Not specified

												115 F/S				high at the outpatient clinic, compared to younger women Cancer-detection rate twice as high in white women aged 50-64 compared to black women aged 50-64 Cancer-detection rate for women 65+ was higher for black wome	

																n than white women Proportion of malignancies detected from biopsies is lowest among physician-referred women aged 50-64 (20%)	
could mobile mammography reduce social and geographic inequalities in breast cancer screeni	2017	Preventive Medicine	Field and clinic	France	Orne, France, far from radiologists' offices (primarily rural)	Women, 50-74	Breast cancer	Mammography	Single screening service	Patients could choose between screening in radiologist's office or mobile mammography unit	Not specitied	Not specified	Conducted in mobilt unit		Screening participation (MM vs RO)	Mobile mammography was associated with a significant increase in individual partici	Did not consider distance from workplace to RO Younger age group over-represented Unable to control the parking place of the MM van, and it was

ng participation?																pation compared to screening in a radiologist's office (OR = 2.9)<br><br>RO participation was lower in deprived and remote areas (<30% participation for distance to RO >20km)<br>7.5% difference in participation rates between the least and most	not randomized

																deprived IRISs (48.09 % and 40.66 % respectively) 16% difference in participation rates between the IRISs (0-5km) to and farthest (30+) from an RO (45.22 % and 29.06 %, respectively) These two differences above were both p<0.001<br><br>Prefer	

																	ence to screen in MM was most conspicuous in age group >70 years (ratio = 22.1) Trends between quintiles of distance and deprivation and RO vs MM were not significant	
mobile breast screening: factors affecting uptake, efforts to	1990	public health	Field	United Kingdom	Areas near Edinburgh (rural)	Women, 50+	Breast cancer	Mammography	Single screening service	Receptionist asked series of questions and data entered directly into microco	Not specified	Not specified	Feasbility questionnaire conducted in mobile unit	Not specified	Not specified	Attendance rates Car ownership Home ownership Acceptabilit	5631 women who attended for screening were screened - As	Not specified

increase response and acceptability										mputer database The questionnaire identified ineligible women Women also asked where they were before visiting the van, how they						y of mobile screening	age increases, screening attendance decreases<br>- 40-44, 33.5% response rate<br>- 45-49, 29% response rate<br>- 50-54, 23.8% response rate<br>- 55-59, 19.1% response rate<br>- 60-64, 14.9% response rate<br><br>Car ownership<br>- the proportion of wome	

																	n who attended screening included a higher proportion of car owners ($p<0.001$)<br><br>Home ownership - the proportion of women who attended screening included a higher proportion of home owners ($p<0.001$)<br><br>The greate	

																	r the distance and the lower the car ownership, the lower the attendance rate The higher proportion of women married and the higher proportion in full-time employment, the greater the attendance rate<br><br>Acceptability of mobile	

																	screening<br>- based on 572 questionnaires (83.3% of those sent out)<br>- 73.3% found the location of van convenient<br>- 75.5% were pleased with the way they were treated<br>- 63.5% had to queue at the van (average waiting time	

																23.5 minutes) - 99.1% would do it again.	
mobile mammographic screening of self-referred women: results of 22,54o screenings	1992	Radiology	Field	United States	New York City (urban)	Women, 35+	Breast cancer	Mammography	Single screening service	Two Transpo 350 mobile mammography machines were used; machines can be wheeled into any fixed site for screening Portable changing rooms were provided if changing rooms were not available A third mammography unit was fixed in a van, providin	Not specified	Van included a registration area, two changing rooms, a mammography room, and dakroom facilities for changing film in cassettes	Conducted in mobile unit Conducted primarily at work site	Memorial Sloan-Kettering	No-shows Equipment failure that requires rescheduling is a greater problem for MM vs fixed site Limited staffing (four technologists work 4-day week with long hours)	Results of screening studies	Results of screening studies - 89% were normal, less than 1% (n=143) had findings indicating biopsy - Biopsies recommended for 288 women - Cancer found in 50 wome	Cost of screening (business costs in NYC raise the cost of an examination

										g a total screening facility Each examination consisted of two views of each breast Films were batch processed daily at the mammography facility of radiology department Computer-generated letter was sent to each participant and designated physician							n (21%)<br><br>15,000 examinations per year are possible Technologist vacation time decreases output by1,600 studies	
an innovative solution to	2017	Int J Surg	Field and	United King	Low socioeconom	Not specified	Colorectal	No screening	No screening per	Colorectal nurse specialist Support	Colorectal nurse specialist	Not specified	Referral process only	Prince Charles Hospital	Not specified	Bowel Bus study characteristi	772 conversations about	Not specified

raise public awareness using a mobile colorectal clinic - the 'bowel bus'			clinic	dom	ic status		cancer	performed	formed	ed by consultant colorectal surgeon psychosocial support services and welfare benefits are also available	trained to high standard			Colorectal nurse specialist trained to high standard Supported by consultant colorectal surgeon psychosocial support services		cs Procedures performed	signs and symptoms of bowel cancer 373 visitors on board the Bowel Bus 10 people referred to the Bowel Screening Programme 244 examined by colorectal nurse specialists - 66 asymptomatic drop-ins - 135 referred to rapid access colore	

																	ctal service by GPs<br>- 43 follow up patients<br><br>- 141 endoscopic procedures performed<br>- No pathology reported in 148 patients<br>- 57 patients had diverticular disease<br>- 23 patients had colorectal polyps<br>- 3 patients had malignant	

																	tumors	
analysis of a decade of skin cancer preventing using a mobile unit in brazil	2019	RURAL AND REMOTE HEALTH	Field	Brazil	Remote areas of Brazil (rural)	Not specified	Skin cancer	Skin cancer screening	Multiple screening services	Not specified	Not specified	Not specified	Conducted in mobile unit Referral process in mobile unit	Barretos Cancer Hospital	Bureaucracy of Brazilian health system	Total number of appointments Number of procedures performed number of referrals made	45872 patients with suspected skin cancer evaluated at the mobile unit from 2004 to 2013 (mean = 4587 per year) 8954 surgical procedures performed - 7098 (15.5%) pathological confirmation of alignancy -	Not specified

																	38774 (84.5% instructed to continue follow-up with GP	
results from lung cancer screening outreach utilizing a mobile ct scanner in an urban area	2024	J Am Coll Radiol	Field and clinic	United States	New York City (urban)	Individuals, 50-80 with ≥20 pack-year smoking history	Lung cancer	SDM, LDCT, tobacco-treatment counseling	Multiple screening services	Patients recruited by walk-ins or after scheduling an appointment		Not specified	Conducted in mobile unit/clinic	NP	Time constraints for physicians Lack of awareness of LCS Concerns about false-positive results and patient resistance (provider-level)	Screening results Incidental findings	Proportion of various Lung-RADS scores assigned to the mobile screening cohort was similar to that of the hospital-based cohort and estimated population prevalence	Non-randomized study design Missing data for both cohorts (retrospective analysis) - more missing race data from mobile cohort Short study duration Many patients unable to follow-up

																of lesions described in each respective Lung-RADS category. LungRADS score 1 and 2<br>- Mobile 88.4%<br>- Hospital 89.8%<br>3<br>- Mobile 5.1%<br>- Hospital 4.7%<br>4A<br>- Mobile 4.6%<br>- Hospital 2.3%<br>4B and	

																4X<br>- Mobile 1.9%<br>- Hospital 3.1%<br><br>Mobile cohort<br>- mean age 60.4 (SD 6.8)<br>- mean pack-year history 38 (25-45)<br>- people in the mobile cohort were more likely to be younger, uninsured, and have a lower-pack-year smoki	

																ng history	
initial results from mobile low-dose computerized tomographic lung cancer screening unit: improved outcomes for underserved populations	2020	Oncologist	Field	United States	North Carolina (rural and urban)	Individuals, 55+ w/ current or recent smoking history, uninsured/underinsnsured patinets	Lung cancer	LDCT	Single screening service	Coach includes waiting area, high-speed wireless internet connection, portable electronic tablet to deliver smoking cessation and health education programs (in english and spanish)<br>Films reviewed by pulmonologists, diagnostic radiologists, and medical oncologists with specific interest in lung cancer	Body Tom CT built into a 35-foot coach	Conducted in mobile unit	Levine Cancer Institute/Atrium Health System		Screening outcomes<br>Patient satisfaction	550 participants scanned total (mean age 61 years, range 55-64)<br>601 pulmonary nodules were identified<br>- 267 participants with LungRADS 1<br>- 183 with LungRADS 2<br>- 62 with LungRADS 3 (11%)<br>- 38 with LungRADS 4 (6.9%)<br>12	Not specified

																	participants with lung cancer<br>- 6 NSCLC<br>- 5 mtastatic MSCLC<br>- 1 metastatic small cell undifferentiated lung cancer<br><br>66% of patients returned for follow-up --> good patient satisfaction	
do mobile units contribute to spatial	2020	Prev Med	Field and clinic	United States	All North Texas counties Low-	Women, 45-75 uninsured seeking	Breast cancer	Mammography	Single screening ser	gathered EHR mammography data, geocode	Not specified	Not specified	Conducted in mobile	Breast Screening and Patient Navigation	Not specified	Travel time to screening	Mobile units deployed 202 times	Some women were excluded because of missing

accessibility to mammography for uninsured women?					income Uninsured, underinsured	mammography			vice	d addresses and mammography facilities			unit/clinic	program (BSPAN)		facility	to 95 locations during study period Total 130 BSPAN mammography locations 6439 total appointments<br><br>4480 received screening w/ BSPAN<br>- 162 (2.9%) received mammo at rural facility<br><br>Travel time to neares	information from HER Did not take into account operating hours of each facility Study only provides population-level information; cannot infer mammography access

																	t facility shorter for women who went to urban mobile vs urban brick-and-mortar (7.64 vs 13.25 min) Travel time to chosen facility longer for women who went to urban mobile vs urban brick-and-mortar (22.32 vs	

																	20.61 min) Travel time to chosen facility long for women who went to rural mobile vs rural brick-and-mortar (30.31 vs 15.94 min) Travel time to nearest facility did not vary in rural settings	

multistrategy health education program to increase mammography use among women ages 65 and older	1992	Public Health Reports	Field	United States	Metropolitan Philadelphia area (urban)	Women, 65+ who had not had mammograms in the previous year	Breast cancer	Mammography	Single screening service	Women selected randomly at each site to participate in baseline survey Women at control sites were randomly selected to p;articipate in baseline interview Women were also offered an opportunity to obtain mammogram on a mobile van that was brought to the retirement community	Not specified	Van contains mammography unit, changing area, waiting area, reception desk	Individuals with mobility issues Lack of awareness Lack of physician recommendation Not all women chose to attend the educational program	Differences between study groups at baseline Baseline beliefs about breast screening Beliefs about breast screening Use of mammography by study group Use of mammography by demographics and other potential confounders Mam	45% obtained mammogram within 3 months in experimental group 12% obtained mammogram within 3 months in control group OR = 6.1 (3.4-10.9)<br><br>Women who attended education session 7.8 times more likely	Demographic imbalance between study groups Cost limitations, could not separate effects of individual intervention components Lack of physician data Limited racial diversity in experimental group

										1 week before the mobile unit's visit, women were invited to attend educational program with video, print materials, group discussion, refreshments						mammography use by retirement community	to obtain mammogram that those who did not attend (OR = 7.8 (4.3-14.1)<br><br>Out of 231 mammograms performed, 11% were abnormal - two breast cancers detected (one metastatic, one early-stage)	
attendees of manchester's	20	Thorax	Field	United Ki	Socioeconomically disadv	Smokers, 55-74	Lung	LDCT, Spirometry	Single screeni	LDCT scanners stationed a	Not specified	Not specified	Conducted in	Nurses Resear	Not specified	Screening adherence	90% of those eligible	No data on those that did not attend the

lung health check pilot express a preference for community-based lung cancer screening	19			ngdom	antaged areas in Manchester (urban)		cancer	equipment	ng service	community retail locations within socioeconomically deprived areas of manchester<br>Led by nurses<br>Lung Health Check at the community site with risk assessment<br>Annyal follow-up LDCT offered to high risk individuals at the same mobile site			mobile unit	ch nurses		Perceived importance of location	attended second screening round<br>74.7% said location was important<br>23% said they would be less likely to attend LCS if it was hospital-based<br>- travel-related 62%<br>- lack of parking 46.3%<br>- parking costs 42.1%<br>- poor public transportatio	second screening round

																	n 31.9%	
mobile mammography in underserved populations: analysis of outcomes of 3,923 women	2013	J Community Health	Field	United States	Jefferson County, KY	Women, 40-75	Breast cancer	Mammography	Single screening service	Team included advanced practice nurse or physician, registered nurses, community health workers, and technical support staff 40-foot mobile unit with digital mammography equipment and exam room for CBEs Traveled to more than 200 locations	advanced practice nurse physician	40-foot mobile unit containing digital mamography equipment and exam rooms for CBEs and counseling	Conducted in mobile unit	advanced practice nurse, physician, registered nurses, community health workers, technical support staff	Not specified	Breast cancers detected Stage at diagnosis abnormal screening results	3923 women screening 4543 mammograms and/or CBEs conducted 31 total breast cancers detected<br><br>Stage at diagnosis<br>- 0: 9.7%<br>- 1: 29.0%<br>- 2: 16.1%<br>- 3: 9.7%<br>- 4: 3.2%<br><br>188 abnormal mam	Retrospective study design Sample bias Potential under reporting of suspicious mammograms due to coding Breast density and BMI not studied

																mammograms (4.1% of all screenings) 236 incomplete mammograms (5.2% of all screenings)	
corporate-sponsored breast cancer screening at the work site: results of a statewide program	1991	Radiology	Field	United States	Philadelphia and Delaware in a large corporation	Women (or employee spouses), 35+	Breast cancer	Mammography	Single screening service	Van dispatched when 20 or more women signed up Radiologists interpreted all mammograms Technologists performed exams Mobile mammography van contained x-ray and	Staff had extensive experience in population-based screening programs Radiologists interpreted all mammograms	Van contained x-tray and film processing equipment	Conducted in mobile unit	Fox Chase Cancer Center for staffing Radiologists	High cost Geographic area covered 47351 sq miles (difficult to obtain f/u data)	Number of screenings conducted Biopsies recommended and performed BC detected Cancer stage	3627 women screened 63 biopsies recommended (1.7%) - 57 biopsies performed 9 cancers detected - 1 palpable - 8	Lower-than expected detection rate - more younger women - more women had recent normal mammograms Expensive and time-consuming to audit Geographic area of 47351 sq miles, difficult to obtain f/u data

										processing equipment Mammograms interpreted using narrative reports and standardized computer-assisted interpretation form Visited 57 sites total Printed health education materials SBE video tape Calendar for tracking breast self-examination							screening-detected Cancer stage - 7/9 (78% were early-stage - 1 cancer Stage II - 1 cancer Stage III	
multiphasic mobile cancer	19	Cancer	Field	United	Lynch, Nebra	Individuals in	Colorectal canc	Mammography, CBE, Pap	Multiple scre	Providers - Physicia	Physicians Dentists	Custom built 60-	Conducted in	Local community	Patient fear Overcrowdi	Total patients screen	1139 total patients	Incomplete physician follow-up Gaps in

screening: a positive approach to early cancer detection and control	72			States	ska (rural)	Lynch, NE	er, prostate cancer, breast cancer, cervical cancer, skin cancer, oral cavity cancer, lung cancer, lymphatic cancer	smear, Proctosigmoidoscopy, Digital rectal examination, Oral cavity examination, skin examination, chest x-ray	ning services	ns conducted proctosigmoidoscopies and pelvic exams<br>- Dentists performed oral cavity exams<br>- RNs assisted in pelvic exams (specifically trained in pelvic anatomy - and pathology)<br>- X-ray technician performed mammogram<br>- Medical social worker, secretary, biostatistician for	RNs (had special training about anatomy of pelvic area)<br>X-ray tech<br>Medical social worker<br>Secretary<br>Biostatistician	foot by 12-foot house trailer<br>Reception area<br>Multiple exam rooms<br>Bathroom<br>Mammography equipment<br>Dental exam station	mobilt unit	hospitals	ng initially<br>Travel burden/lack of transportation in rural areas<br>High cost<br>Apathy toward screening	ed<br>Cancers detected<br>F/U rate	screened<br>- 2 cases colon cancer<br>- 1 case prostate cancer<br>- 1 case breast cancer<br>- 1 case lung cancer<br>- 1 case lympatic cancer<br><br>Of the 295 patients with significant findings, 47.1% followed up w/ physician	skin cancer diagnosis<br>Long-term outcomes not yet measured

										support Equipm ent - Mammo graphy equipme nt - Dental exam room<br><br>Visited sites near commun ity hospital s								
effecti veness of a mobile mamm ograp hy progra m	2 0 1 7	AJR Am J Roent genol	Fiel d and clin ic	Un ite d Sta tes	Charle ston, SC and 8 neighb oring counti es (urban and rural)	Women, low- income, uninsure d, minority populati ons	Brea st canc er	Mamm ograph y	Sin gle scre eni ng ser vic e	Technici ans perform mammo grams Radiolo gists interpret images Clinical staff responsi ble for commun ication Mobile unit based in commun ity locations (e.g. Walmart locations	Mamm ograph y techno logists, Radiol ogists	Not specif ied	Cond ucted in mobil e unit	Medic al Univer sity of South Caroli na	Not specifi ed	BC detecti on rate Abnor mal mam mogra m rate Follo w-up compl etion Stage of BC at Dx	55 total cases detect ed - 38 from perma nent clinic (69.1%) - 17 from mobil e clinic (30.9%) 28 early- stage cases at the	Selection bias Loss to follow-up Does not consider data captured outside MUSC health system Limited generalizabi lity

										, outlet malls) Unit operated in Charleston and 8 surrounding counties							clinic (72.7% of 387 early-stage cases from the mobile unit (41.2% of 17) Patients who received biopsy after imaging - Clinic 29.7% - Mobile unit 15.0%	
mobile breast screening services in australia: a qualitative exploration of percep	2024	Australian Journal of Rural Health	Field	Australia	Rural and Remote Areas of Australia (rural)	Women, 75+	Breast cancer	Mammography	Single screening service	Delivered through BreastScreen Australia program Mobile units visited every 2 years Women	Not specified	Not specified	Conducted in mobile unit	Not specified	Difficulty traveling 2/2 limited mobility or lack of transport options	Perceived value of screening Satisfaction Frustration	Participants saw breast screening as important, beyond age of 75 Women said they	Women who chose to not participate were more difficult to reach to discuss their decision-making

tions and experiences among rural and remote women aged ≥75 years										75+ were not actively invited, but could self-refer							would continue to attend mobile service Women felt excluded because they were no longer invited to screen after 74	
acceptability of mobile mammography among community-dwelling older women	1997	J Am Geriatr Soc	Field	United States	City of Los Angeles (urban)	Women, 60-84	Brease cancer	Mammography	Single screening service	Free, on-site mobile mammography Conducted at 12 community meal sites CBE by NP, breast self-exam instruction, mammogram	Nurse practitioner	Not specified	Conducted in mobile unit	City of Los Angeles Area Agency on Aging	Ethnic/cultural-specific barriers that prevent women from receiving mammogram Lack of physician	Mammography acceptance rate beliefs and intentions related to breast cancer screening Functional	Mammography acceptance rates - 255 women volunteered - 148 women eligible - 85 (57%) women	Convenience sample was used --> limited generalizability Influenced decision-making by site directors Free mammography as a potential confounder Limited ethnic and geographic

															referral,	status score	accepted<br>- 63 (43%) women accepted<br><br>Logistic regression equation preducting acceptance of mobile mammography<br>- HMO membership (OR = 0.30, CI: 0.12-0.73)<br>- Asian american (OR = 11.8, CI:3.2, 43.1)<br>-	representation

																	Overdue or never had mammogram (OR = 0.37, CI: 0.13-1.1)<br>- Believes mammography be performed frequently (OR = 2.5, CI: 1.1-5.6)<br>- Single or never married (OR = 0.13, CI: 0.02-1.1)<br>- Willing to accept mammography if	

																	rec by physician (OR = 3.8, CI: 0.83-17.0)	
mammograms on-the-go - predictors of repeat visits to mobile mammography vans in st louis, missouri, usa: a case-control study	2015	BMJ Open	Field	United States	St. Louis, Missouri (urban and rural)	Women who received a mammogram on the van between April 2006 and March 2014	Breast cancer	Mammography	Single screening service	Mammograms delivered via mobile mammography van Served women who qualified for financial assistance April 2006 to March 2014 Mammograms offered at little to no cost	Not specified	Not specified	Conducted in mobile unit Conducted primarily at work site	Alvin J Siteman Cancer Center at Barnes-Jewish Hospital	Rural locations had less repeat visits by study participants	Repeat use of mobile mammography sites Adherence to screening guidelines Patient-reported mammography experience	25.3% of women had repeat visits to the MMV - Women who were 50-65, uninsured, or African-American had higher odds of a repeat visit to the mmv compared with women who were	Data not generalizable to populations who screening guidelines differ from ACS guidelines Did not investigate reasons why women missed routine screening

																	40-50, insured, or Caucasian (OR = 1.135, CI: 1.013-1.271; OR = 1.302, CI: 1.146-1.479; OR = 1.281, CI: 1.125=1.457) - The odds of having a repeat visit were lower among women who reported a rural zip code or were unemployed	

																	compared with women who had a suburban zip code or were employed (OR = 0.503, CI: 0.411-0.616; OR = 0.868, CI: 0.774-0.972)	
mobile versus fixed facility: latinas' attitudes and preferences for obtaining a mammogram	2018	Journal of the American College of Radiology	Field and clinic	United States	Washington (urban)	Women, latina, 40+	Breast cancer	Mammography	Single screening service	Mobile units in community settings Spanish-speaking personnel	Spanish speaking staff members for mobile mammographic services deployed in Latina	Not specified	Conducted in mobile unit/clinic	Department of Radiology, University of Washington, Seattle Cancer Care Alliance	Language barriers	Preference for mammography service location Perception of quality between servic	52.3% of Latinas preferred obtaining a mammogram at a fixed facility compared with havin	Separate sample for quantitative and qualitative aspects Selection bias Cross-sectional data

											communities					e types Predictors of service location preference Changes in perception after using mobile services	g no preference (46.3%) and preferring mobile mma mographic services (1.7%) - 15.6% of participants were concerned about privacy and comfort - 10.6% were concerned about general quality - 51.3% felt the qualit	

																	y between fixed site and mobile was equal<br>- 33.1% felt the quality at the clinic or hospital was better<br>- 0.4% believed the quality at the mobile mammographic service was better<br><br>Women with a history of a prior mam	

																	mogram had 1.7 times greater odds of expression no location preference for obtaining a mammogram or preference for obtaining a mammogram using a mobile mammographic service compared to women with no histor	

																	y of a prior mammogram.	
an intervention to increase mammography use by korean american women.	2004	Oncology nursing forum	Field	United States	Southern California (urban)	Women, Korean, 40-75	Breast cancer	Mammography	Single screening service	Peer-Group Educational Program (1 hour)<br>- delivered in Korean at Korean churches<br>- integrated cultural values<br><br>Access to mobile mammography<br>- low-cost or free mobile mammograms offered on-site at churches one week	Peer educators<br>Nurse educator<br>Mobile mammography staff	Not specified	Conducted in mobile unit	Azusa Pacific University	Cultural beliefs about cancer<br>Language barriers<br>Limited access to affordable services	Mammography use<br>Breast ccancer screening knowledge<br>Breast cancer screening attitudes	Mammography use<br>- 87% of women in "Let's Talk" received mammogram<br>- 72% in the mobile mammography-only group<br>- 47% in the control group<br><br>Breast cancer screening knowledge<br>-	Non-random sample of participants<br>Limited generalizability<br>Self-reported mammography use<br>Potential response bias<br>Unmeasured variables

										after baseline survey<br><br>Three group-design<br>- Experimental group<br>- Comparison group<br>- Control group							Significant improvement knowlegde in the intervention group (63.66 --> 89.42 (p<0.001))<br><br>Breast cancer screening attitudes<br>- Overall attitude score improved from 65.3 to 79.8 (<0.001)	
the performance of mobile screening	2018	Cancer Causes and Control	Field	Brazil	Northern Sao Paulo state	Women, 40-69	Breast cancer, cervical	Mammography, Pap smear	Multiple screening ser	4 total mobile screening units (2 with digital mammo	Radiologic Technicians Radiologists Comm	2 units equipped with digital	Conducted in mobile unit	Division of Cancer Epidemiology, McGill	Geographic distance Recall rate higher	Mammography screening coverage	Screening coverage<br>- 54.8% (223,4	No data on individual-level risk factors Limited data on follow-up

units in a breast cancer screening program in brazil					(urban)		cancer		vices	graphy, 2 with film-screen mammography<br>Can serve 60 patients per day<br>Radiology technician, radiologists, community health agents, fixed-site clinical staff	unity health agents	mammography<br>2 units equipped with analog film-screen mammography<br>Mammography room where exam was conducted<br>Adjacent exam room to allow for Pap smears		University	than European guideline<br>Lack of double reading of mammograms	Abnormal mammogram rate<br>Follow-up completion for abnormal screens<br>Diagnostic outcomes	67 women across 108 municipalities screened)<br><br>Abnormal mammogram rate - 12.25% (15,025 with abnormal results out of 122,634 total screened)<br><br>Follow-up completion rate - 92.35% (13,879 who completed f/u	timing<br>No cost analysis<br>Limited generalizability

																	after abnormal mammogram) Diagnostic outcomes - BIRADS 1 or 2: 79.2% - BIRADS 3: 9.3% - BIRADS 4 or 5: 11.5%	
availability of prior mammograms affects incomplete report rates in mobile screening mamm	2018	Breast Cancer Research and Treatment	Field and clinic	United states	Rural, underserved	Women, 41+	Breast cancer	Mammography	Single screening service	Great Plains Mobile Women's Health Unit Served 18 rural and small urban Indian Health Service clinics across 4 US	Radiologists certified under the Mammography Quality Standards Act who specialize in breast	Contained a GE Medical Systems 2000 D, FFDM unit	Conducted in mobilt unit/clinic	IHS clinics Comprehensive cancer center	Lack of previous mammogram for comparison Geographic isolation Limited patient	BIRADS assessment categories Rate of prior mammogram availability	2640 total mammograms analyzed 2179 unique patients BIRADS 0: 11.7% Priors availa	Retrospective nature Smaller set of mobile mammography cases

ography										states Digital FFDM unit<br>- images were transmitted by satellite, internet, or mail to a fixed-site comprehensive cancer center for interpretation<br>Radiology technologists<br>Radiologists	imaging				history and documentation<br>Technological limitations		ble<br>- BIRADS 0: 7.8%<br>Priors unavailable<br>- BIRADS 0: 13.1%<br><br>aOR = 0.53, CI: 0.39-0.72	
adherence to screening among american indian women accessing a mobile mammograp	2021	Acad Radiol	Field	United States	Rural	Women, 42+	Breast cancer	Mammography	Single screening service	Traveled to 24 Indian Health Service clinic sites (5028 miles per year)<br>Unit remained 3-5 days at	Not specified	Not specified	Conducted in mobile unit	Indian Health Service c Mobile Women's Health Unit	Infrequent scheduling of mobile van<br>Delays in reporting results<br>Inaccessibility	Adherence to repeat screening<br>Predictors of screening adherence	1117 women eligible for repeat screening<br>42.3% repeat mammogram within 27	possibility of missing or inaccurate information of the presence of or dates of prior mammography in records of the NMD, which would

hy unit										each clinic site Unit equippe d with digital mammo graphy system Data stored on CDs and mailed to a breast imaging division at academi c center for interpret ation					durin g winter month s Lack of contin uity infrast ructur e Geogr aphic barrie rs		month s Wome n living farther from the screen ing site had lower odds of return ing for repeat mam mogra m (p<0.0 01) Older age was associ ated with a higher adher ence (p = 0.001) Screen ing adher ence varied signifi cantly by clinic	result in lower adherence to screening rates Variety of reasons that may affect adherence were no evaluated (i.e. houdhold income, education, comorbidie s, availability of transportati on)

																	site (p<0.001)	
providing free mammography screening to uninsured muslim women in south florida	2023	Journal of Health Care for the Poor and Underserved	Field	United States	South Florida (rural and urban)	Women, Muslim from South fFlorida	Breast cancer	Mammography	Single screening service	free-standing mobile mammography van that provides free screening mammograms to eligible uninsured residents of Miami-Dade county No male staff Medical student volunteers 3D mammography technology 3 visits were conducted at the Islamic Center of	Community and religious leaders Clinical staff on van No male staff	Not specified	Conducted in mobile unit	The Imam of ICGM Female community leaders Mosque	Women expressing hesitation about being exposed during screening Language barriers Lack of knowledge and mistrust Low prior screening rates	Mammographic screening uptake Prior screening history BIRADS	33 women received a mammogram 30.3% of women never had a mammogram before BIRADS0: 58% BIRADS 2: n = 6 BIRADS 3: n = 3	Small sample size (33 women) Single community focus limits generalizability

										Greater Miami								
telemedicine system using computed tomography van of high-speed telecommunication vehicle	2001	IEEE Transactions on Information Technology in Biomedicine	Field	Japan	Rural	Individuals, 50+, chronic smokers	Lung cancer	LDCT	Single screening service	Van contained Spiral CT scanner, high-speed telecommunication system, video conferencing system Radiologic technologists and physicians; radiologists at tertiary care hospitals Rural lung cancer screenings; emergency care at winter sports events; home-	Radiologic technician Radiologist	Van equipped with spiral CT scanner for LDCT Asynchronous Transfer Mode with real-time video conferencing for immediate specialist interpretation at tertiary hospitals	Conducted in mobile unit	Specialists at tertiary hospitals Telecommunication Advanced Organization of Japan Shinshu University Hospital	High costs Transmission speed limitations Requirement for heating systems at the Nagano Winter Olympics	Number of lung cancer cases detected Treatment outcomes Hospitalization duration Medical treatment costs	19,117 individuals screened total 75 cases of lung cancer detected 0.39% detection rate Diagnosed individuals treated with partial pneumonectomy and video-assisted thoracic surgery Hospitalization duration	High operational costs Data transmission delays

										care settings							- In 1997, 27.7% shorter stay for those diagnosed via mobile CT compared to those diagnosed in hospital<br>- In 1998, 25.6% shorter stay<br>Treatment costs<br>- In 1997, Mobile CT group had 45.1% of hospital group's treatment costs	

																	- In 1998, mobile CT group had 35% of hospital group's treatment costs	
a mobile colonoscopic unit for lynch syndrome: trends in surveillance uptake and patient experiences of screening in a developing country	2013	Journal of Genetic Counseling	Field	South Africa	Western and Northern Cape Province (rural)	GESC patients, 18+, documented to have MMR gene mutation	Lynch syndrome	Colonoscopy	Single screening service	Colorectal surgeons, gastroenterologists, nurses, genetic counselors Sedatives for procedure (dormium, pethidine) Mobile colonoscopic unit to remote sites, audiovisual systems	Colorectal surgeons, gastroenterologists, nurses, genetic counselors Trained to manage individuals with Lynch syndrome	Not specified	Not conducted in mobile unit Van for annual outreach only	Colorectal surgeons, gastroenterologists, nurses, genetic counselors Genetic and Endoscopic Surveillance Clinic (GESC) Ambulanace Transport Services	Pregnant patients, work obligations, family obligations Uncertainty about screening date/frequency of screening Colon preparation difficulties Toilet facility	Adherence to recommended colonoscopy Patient experiences	93% of participants had attended at least one colonoscopy Fewer than 25% had perfect adherence Self-reported adherence significantly overestimated	qualitative research cannot comprehensively reveal all practice implications participants may have provided answers construed as appropriate rather than answers reflecting their true attitudes

										for patient education, ambulance transport, hospital records and database systems Mobile program was conducted yearly in four centers					limitations Transportation concerns		compliance Patient experiences - 48.8% described colon preparation as unpleasant - 18.8% only had outdoor toilets, making preparation difficult - 33.8% described procedure as fine - 28.8% described procedure as	

																	painful - 94.5% received colonoscopy results - 90.4% understood their results - 90% knew when their next colonoscopy was scheduled	
breast cancer screening in older black women	1994	Cancer	Field	United States	Erie County, New York (rural)	Black women, 65+	Breast cancer	Mammography, CBE	Multiple screening services	Driver, outreach workers, nurses, physicians, community volunteers BSE instruction using silicone breast models CBE	Physicians, nurses, outreach workers, community volunteers, driver All staff completed 6-week	34-foot mobile education and screening van included classroom with seating for	Referral process only	Breast Cancer Detection Project Department of Cancer Control and Epidemiology, Roswell Park Cancer	Mammography not provided in mobile clinic Long wait times Women confusing mam	Participation BSE practices Mammogram history Engagement	99% of 271 older black women participated in breast health education sessions 75% receiv	Limited generalizability Smalle sample size Reliance on self-reported data Lack of variation within older black female population

										Needs assessments and referrals for mammograms at external facilities ~4 programs per month for older women	training program before participating in community activities	15-18 women examination room library for educational materials		Institute, Buffalo, New York	mogram with other tests Education and literacy challenges		ed CBE after education session 13% followed through with referral to get mammogram 20% of women reported practicing BSE regularly 47% practiced occasionally 33% never practised BSE 57% reported having receiv	

																	ed at least one mammogram in their lifetime	
a proximity care paradigm for cancer screening with a mobile multi-screening unit in inner areas	2024	2024 IEEE International Conference on Pervasive Computing and Communications Workshops and other Affiliated Events, PerCom Workshops 2024	Field and clinic	Italy	Mountainous inner area in Tuscany (rural)		Breast cancer, cervical cancer, colorectal cancer, melanoma	Mammography, gynecological exam and sample collection, digital video dermatoscopy	Multiple screening services	Screened for breast cancer, cervical cancer, colorectal cancer, and melanoma radiology technician, obstetrician, nurse Mammography unit, digital dermatoscopy system, gynecologic exam table, 32L refrigerator, four	Radiology technician, obstetrician, nurse All staff underwent 2 hour training program	FIAT ducato insulated and waterproof 0.3mm lead shielding in mammography room Air conditioning Mammography instrument, digital dermatoscope, gynec	Conducted in mobile unit	Health care professionals ASL Toscana Nord Ovest ISPRO Municipalities in the Valle del Serchio	Internet issues Complex data across multiple systems made it difficult to integrate with hospital systems Limited space and equipment constraints	Procedures performed Dermoscopy participation rate	>500 citizen screened >650 procedures performed ~35% of patients opted for melanoma screening	Short implementation period No cost-benefit analysis

										work stations Visited 5 municipalities 3-4 days per week, 6 hours per day		ological exam table, four work stations						
do appalachian women attending a mobile mammography program differ from those visiting a stationary mammography facility?	2013	J Community Health	Field and clinic	United States	Appalachia (rural)	Women, 40+	Breast cancer	Mammography	Single screening service	Bonnie's bus serves multiple counties in WV 60% of Bonnie's Bus overlaps with stationary services	Not specified	Not specified	Conducted in mobile unit	Mary Babb Randolph Cancer Center West Virginia University Health care	Not specified	Adherence Perceptions and knowledge Routine screening behavior Program reach	1161 mobile mammography users 1104 stationary facility users Adherence ($p<0.0001$) - Mobile unit 48.2% adherent, 51.9% non-adherent - Stationary 92.3% adher	Low response rates Differences between respondents and non-respondents Limited generalizability

																	ent, 7.7% non-adherent Knowledge and perceptions (p<0.0001, p=0.0004, p=0.0004) - Mobile unit 47.1% high knowledge scores - Stationary 31.3% high knowledge scores - Mobile unit 12.1% perceived high 5-year BC risk -	

																	Stationary 15.6% perceived high 5-year BC risk<br>-<br>Mobile unit 13.9% perceived high lifetime risk<br>-<br>Stationary 17.8% perceived high lifetime risk	
effectiveness, safety and acceptability of 'see and treat' with cryotherapy by nurses in a	2007	British Journal of Cancer	Field	India	Dindigul District, Tamil Nadu, South India (rural)	Women, 30-59 with an intact uterus and no past hx of cervical neoplasia	Cervical cancer	Visual inspection with acetic acid (VIA), coloscopy, cryotherapy	Multiple screening services	Clinics were set up in village primary health centers, municipal offices, schools, women's club buildlings	8 nurses, each with 3 years of nursing education, attended 3-week intensive training<br>Gynecologist, clinical oncologist, or GP was always present at the field clinics		Conducted in mobile unit	IARC (for training)<br>PSG Institude of Medical Sciences and Research (for	Not specified	Treatment uptake<br>Histology findings<br>Follow-up and cure rates<br>Accep	2513 women total were offered cryotherapy after screening<br>-<br>74.8% of wome	Loss to follow-up<br>Overtreatment

cervical screening study in india										8 nurses trained in VIA, coloscopy, biopsy and cryotherapy (underwent 3-week intensive training program)<br>- doctor always present at the clinics to supervise<br>Colposcope, cryotherapy equipment<br>Biopsy tools<br>Sanitary pads<br>Metronidazole and doxycycline for use up to 5 days after tx			pathology)		tability	n accepted and received tx on the same day as screening and bx<br>- 88.8% of women with histologically confirmed CIN received cryotherapy<br><br>Histology findings<br>- 55.6% had confirmed CIN (CIN 1: 1242; CIN 2: 112; CIN 3;	

										Paracetamol for mild pain post-treatment							43)<br><br>Follow-up and cure rates<br>- 1026 women (73% returned for f/u at 6-56 months (mean = 27)<br><br>Acceptability<br>- high same-day tx rate (74.8%) and low refusal/dropout suggest strong acceptability	
implementing lung cancer screeni	2019	Thorax	Field	United King	Manchester (urban)	Ever smokers, 55-74	Lung cancer	LDCT	Single screening	mobile units next to local shoppin	radiologists with specialization	Not specified	Conducted in	National Health Service Wythe	Not specified	Detection of Lung Cancer	46 lung cancers detect	One death occurred within 90 days of surgery

ng: baseline results from a community-based 'lung health check' pilot in deprived areas of manchester				dom					service	g centers in "deprived areas of Manchester" NHS consultant radiologists Mobile CT scanner, spirometry, respiratory symptom assessment, lung cancer risk estimation	s in thoracic imaging		mobile unit	nshawe Hospital		Treatment intent Participation and uptake	ed among 1384 individuals who underwent LDCT (3.3%)<br>- 65.2% were stage I<br>- 87.0% were stage I or stage II<br><br>79% of diagnosed cases were managed with curative intent<br><br>81 individuals referred to the lung cancer clinic	Not a clinical trial

																	- 39 false positi ves (48.1%)<br><br>2541 people attend ed initial lung health checks - 1376 (54.2%) eligibl e for LDCT - 1384 scans perfor med	
increas ing mamm ograp hy uptake throug h acade mic- comm unity partne rships targeti ng immig	2 0 1 8	WMJ	Fiel d	Un ite d Sta tes	Milwa ukee (urban)	Women, 40+	Brea st canc er	Mamm ograph y	Mu ltip le scre eni ng ser vic es	Medical oncologi sts, licensed internist, nurse practitio ner, commun ity health workers, translato rs, voluntee rs Mobile	Medic al oncolo gists, license d interni st, nurse practiti oner, comm unity health worker s, transla	Not specif ied	Cond ucted in mobil e unit Referr al proces s in mobil e unit	Wisco nsin Well Woma n Progra m Multili ngual staff	Lack of health insura nce Lack of mam mogra phy aware ness Lack of knowl edge of	Mam mogra m uptak e Follo w-up outco mes Partici pant satisfa ction with interv ention	Mam mogra m uptak e after works hop - Privat ely insure d wome n had 100% uptak e after	Not randomized trial --> no control group Had to exclude 108 of 493 who opted out of completing the survey 2/2 literacy reasons Patient confidential ity prevented

rant and refugee communities in milwaukee										mammography unit, educational materials<br>Workshops held monthly for 24 months; quarterly mobile mammography visits aligned w/ workshops	tors, volunteers				benefits of mammography<br>Non-English speakers<br>Fear of breast cancer dx<br>Forgetting to schedule mammography<br>Transportation		workshop (n = 113)<br>- Uninsured women (n = 75) had 80% uptake<br><br>12 women required additional diagnostic imaging<br>1 woman dx w/ bc; successfully completed treatment<br><br>Participant satisfaction (N = 374)<br>- 73%	researchers from aiding in survey completion --> missing data

																	found workshop "extremely informative" - 74.6% rated presentation as "extremely clear" - 74.9% found the group friendly and helpful	
breast cancer screening in underserved women in the bronx	1999	J Natl Med Assoc	Field	United States	Bronx (urban)	Women, ≤40	Breast cancer	Mammography	Single screening service	Two 33-foot mobile mammography vans Equipped with Mammair DC mammography systems Trained health professionals	Not specified ("trained health professional")	33-foot mobile mammography van Registration area Two changing rooms	Conducted in mobile unit	Montefiore Medical Center Women's Outreach Network	Lack of health insurance Administrative delays Social/ educational barriers Mistrust and Lack	Cancer detection rate Diagnostic outcomes Mammography results Treatment access	Of the 1962 women screened… 25 breast cancers were diagnosed (12.9 per 1000 women)	Absence of funding for treatment of cancer detected

										perform CBE Patient navigators handled administrative work Radiologists interpreted films following day		Mammography room Darkroom facilities			of awareness		49 biopsies performed (PPV: 51%) Mammography results - 81.4% BIRADS 1 - 7.2% BIRADS 3 - 1.8% BIRADS 4 - 0.05% BIRADS 5 - 5.3% incomplete	
patient compliance in mobile screening mammography	1995	Acad Radiol	Field	United States	North Carolina (rural)	Women served by mobile mammography van	Breast cancer	Mammography	Single screening service	Operated in 10 North Carolina rural counties Perform mammography for asymptomatic women only	Radiologists (board-certified)	Not specified	Conducted in mobile unit	Carolina Screening Mammography Program	Limited access to medical facilities Transportation barriers to reach	Mammographic abnormalities Follow-up recommendations Compliance with	5575 women screened 1005 women with abnormalities - 35.5% requir	No patient tracking system Incomplete follow-up data Self-referral Missing sociodemographic data

										Returned to each location at least once per year<br>3-5 days a week, 3 weeks per month<br>Radiologists to interpret mammography<br>Van equipped with ACR-accredited mammography machine					f/u sites<br>Financial barriers<br>Low physician support	follow-up	ed immediate follow-up<br>- 18.4% 6-month follow-up<br>- 46.1% require 1-year f/u<br>Compliance rates<br>- 47.1% returned for follow-up (compliant)<br>- 52.9% were noncompliant	
outcomes of recommendations for breast biopsi	1998	Public Health Rep	Field	United States	Suffolk County (rural)	Women in Suffolk county	Breast cancer	Mammography	Multiple screening ser	Operated by Suffolk County Department of health services	Registered nurse<br>Radiology technician<br>Clerica	Not specified	Conducted in mobile unit	Suffolk County Department of Health	Patient fear<br>Limited biopsy uptake<br>Physic	Cancer detection rate<br>Positive biops	4369 mammograms performed on 4157	Potential underestimation of bx and cancer detection rates

es in wome n receivi ng mamm ogram s from a county health van									vic es	Visited county health centers and commun ity locations 6 days per week Register ed nurse, radiolog y technicia n, clerical worker Mammo grams interpret ed by radiolog ists CBE, Two-view screenin g mammo gram, instructi on on breast self-examina tion Mammo graphy	l worker Radiol ogist (board - certifie d)			Service s	ian coordi nation Incom plete infor matio n from physic ians	y rate Cance r detecti on by age group Follo w-up compl iance	wome n 59 wome n referre d for biopsy (1.4%) 34 bx perfor med (0.78%) - 14 cancer s detect ed (41.2%) - 20 benig n bx Cance r detecti on rates - 14 cancer s amon g 4349 mam mogra ms (0.32%) - Wome n ≥50, 10	

										instrument							cancers (0.36% detection rate) - Women ≤50, 4 cancers (0.25% detection rate) From biopsies - Women ≥50, 10 cancers from bx (53%) - Women ≤50, 4 cancers from bx (27%)	
mobile mammography utilization and	2025	clinical breast cancer	Field	United States	Rural and urban		Breast cancer	Mammography	Single screening ser	Mammography machines for digital breast tomosyn	Not specified	Not specified	Conducted in mobile unit	American College of Radiology	Economic sustainability Medicare	Adherence to mammography Demo	2630670 screening-eligible 130206	Only Medicare fee-for-service beneficiaries studied Underrepor

breast cancer screening adherence among medicare fee-for-service beneficiaries									vice	thesis Imaging performed in mobile unit and interpreted by radiologists remotely					reimbursement	graphic differences	9 (49.5%) had ≥1 mammogram during enrollment 4973 women (0.4%) received at least one mobile mammogram; 99.6% used facility-based mammography<br><br>Adherence - Among women with ≥1 mammogra	ting/underestimation of MM use Study did not look at access or patient barriers

																	m<br>- For FBM, women went average 7.0 years without a mammogram (SD = 4.5)<br>- For any MM, women went average 6.7 years without a mammogram (SD = 4.3); p<0.001<br><br>Demographic differences<br>- Rural residents: OR =	

																	3.07 (2.86-3.29) - Lower income communities: OR = 1.43 (1.31-1.55)	
mass screening for lung cancer with mobile spiral computed tomography scanner	1998	the lancet	Field and clinic	Japan	Rural and urban	Individuals, 40-74 who underwent annual chest radiography and cytological assessment of sputum	Lung cancer	Spiral computed tomography	Single screening service	LDCT scanner in a van serving multiple locations in 29 municipalities in Japan Radiologists interpreted scans; technologists sent by Antituberculosis Association Nagano Prefecture Branch for operating mobile	Technologists sent by Antituberculosis association Nagano Prefecture Branch Radiologists	Spiral CT scanner installed in van (CT-W950 SR)	Conducted in mobile unit	Antituberculosis Association Nagano Prefecture Branch Shinshu University School of Medicine	Not specified	Lung Cancer Detection Rate Stage and size of cancers Comparison w/ conventional screening methods	5483 total individuals screened 19 cases 0.35% detection rate<br><br>17 mm mean size of detected lesions (6-47mm)<br><br>Among 19 cases detected, - 14	Poor detection rate for adenocarcinomas of the lung

										unit Leaflets delivered to all homes; participants were self-selected volunteers							were identified as suspicious on CT - 3 were benign but suspicious - 2 were indeterminate	
implementation of an integrated lung cancer prevention and screening program using a mobile computed tomography (ct)	2022	Cancer Control	Field	Brazil	Barretos, Brazil (urban)	Individuals, 55-74 with 30+ pack-year smoking history	Lung cancer	LDCT	Single screening service	Truck trailer with Ct Scanner Doctors, nurses, dentists, pharmacists Radiologists interpreted LDCT scans Navigator staff for participant assessments and f/u coordinations	Doctors, nurses, dentists, pharmacists underwent 16-hour training provided by CRATOD Within mobile unit: radiologists, navigator staff,	Air-conditioned CT room GE Healthcare Optima 540 CT scanner Control room Working facilities Entry access via ladde	Conducted in mobile unit	CRATOD Barretos Cancer Hospital	Mobile unit couldn't travel long distances (Barretos only) Lack of reliable information to identify participant population Smoki	LDCT screening results (Lung RADS results) Smoking cessation success Screening access	233 high-risk individuals screened (52.8% had high or very high nicotine dependence) Lung RADS results: - 1 or 2: 83.7%	Bias 2/2 non-representative study population

unit in brazil										Interventional radiologists performed bx when necessary<br>Nicotine patches and bupropion hydrochloride for smoking cessation tx<br>Mobile unit operated within Barretos city boundaries	interventional radiologists	r or hydraulic elevator			ng cessation resources inconsistent across facilities		- 3: 7.3%<br>- 4A: 7.7%<br>- 4B or 4X: 3.0%<br><br>Of those enrolled in the smoking cessation program, 27.8% successfully quit after 1 year<br><br>Screening access<br>- 55.8% accessed through opportunistic encounters<br>- 44.2% referred from	

																	primary care-base smoking cessation programs	
mammography-based screening program: preliminary results from a first 2-year round in a brazilian region using mobile and fixed units	2012	BMC Womens Health	Field and clinic	Brazil	Rural	Women, 40-69	Breast cancer	Mammography	Single screening service	1 mammography machine in a mobile unit - Capable of 40 exams/day (daily average = 26.3) Fixed unit at Barretos Cancer Hospital - capable of 120 exams/day (daily average = 18.6)<br><br>Radiologists interpreted mammo	Radiologists	quipped with Senograph 700 T mammography machine	Conducted in mobile unit/clinic	Barretos Cancer Hospital	Lack of national registry to track screening Private health insurance --> opt out Lack of community intervention experience low adherence of physicians that worke	Coverage Access Utilization Cancer detection	33.1% of eligible underwent mammography 58.6% of women used the MU for mammography 41.4% of women used the FU for mammography MU performed 26.3	Failed to separate symptomatic from asymptomatic women High demand for screening not equal to health policy prioritizaion Low bx yield 2/2 low radiologist training

										grams and performed mammography in the MU					d in the municipalities<br>lack of organization in the Brazilian health system<br>cultural concerns that the examinations may identify cancer		exams/day<br>FU performed 18.6 exams/day<br>76 cancers detected (4.2 cases per 1000 exams)<br>- 43.4% were early-stage<br>- MU identified 39.1% early stage, 17% late-stage<br>- FU identified 48.6^ early-stage, 14.3% late-stage	

participation in community-based lung cancer screening: the yorkshire lung screening trial	2022	Eur Respir J	Field and clinic	United Kingdom	Leeds (urban)	Individuals, 55-80	Lung cancer	LDCT	Multiple screening services	Mobile units located at 11 community sites Research nurse, senior clinical trial assistant LDCT scanner, spirometry, CO monitors	Research nurses, senior clinical trial assistants	Not specified	Conducted in mobile unit	The Royal Infirmary	Low particpation among people who currently smoke Low participation among those from more deprived areas Older individuals less likely to attend	Attendance Screening completion Impact of smoking on participation	22815 of 44943 invited individuals responded ot the telephone triage 86.8% attended appointment after being offered one 99.7% eligible individuals underwent LDCT People who currently smoked were 56% less likely to respond to the	Not specified

																	invitation (aOR = 0.44 (0.42-0.46)) and 27% less likely to attend the LHC (aOR = 0.73 (0.62-0.87)) People in the most deprived areas were 42% less likely to responde (aOR = 0.58 (0.54-0.62)) and 22% less likely to attend the LHC	

																	(aOR = 0.78 (0.62-0.98))	
mobile screening unit (msu) for the implementation of the 'screen and treat' programme for cervical cancer prevention in pune, india	2021	Asian Pac J Cancer Prev	Field and clinic	India	IN and around Pune city (urban and rural)	Women, 30-60	Cervical cancer	VIA	Single screening service	290 outreach clinics Exam bed, light, wash basin, clock, air-conditioning, thermal ablation devices Medical officer, community coordinator, counselor, data entry operator, nurses, drive WhatsApp used for coordination	Nurses and HCPs trained to perform VIA and thermal ablation Medical officer	Front compartment with seating and storage Back compartment with exam bed, light source, platform for instruments, wash basin, generator	Conducted in mobile unit	Local NGOs, self-help gorups, women's organizations WhatsApp for communication	Low referral compliance	Treatment outcomes Treatment w/ thermal ablation Side effects Colposcopy outcomes	10925 women screened total 717 women tested positive via VIA 304 women treated with thermal ablation 3.6% experience minor side effects Colposcopy outcomes - CIN 1: 7 women (8.3%) - CIN	High loss to f/u No investigation into noncompliance Potential underdiagnosis in those not followed up

																	2: 2 women (2.4%) - CIN 3: 9 women (10.7%) - Invasive cervical cancer (2.4%)	
the impact of accessible cervical cancer screening in peru- the día del mercado project	2015	J Low Genit Tract Dis	Field and clinic	Peru	Andes Mountain villages and Cusco (urban and rural)	Indigenous Peruvian women, 16+	Cervical cancer	Pap test	Single screening service	Mobile clinics were tents/existing structures at marketplaces Clini	Not specified	Not specified	Conducted in mobile unit	CerviCusco	Privacy concerns, lack of knowledge about pap tests, Distance to screening site, embarrassment	Access to cervical cancer screening Patient-report acceptability Patient understanding	4560 women participated in screening Women seen in tents were more likely to report that it was easier to get a Pap test (98.7% compa	Data on f/u compliance not yet available Convenience sampling --> selection bias Limited information on actual clinical outcomes

																	red to those in buildings (96.8%) or Cervi Cusco (98.0%) Larger proportion of women screened in tents never had a Pap test before (58.3%) compared to those seen in buildings (63.1% or at Cervi Cusco (64.3%) 67.0% of wome	

																	n in tents felt the market was a good place to receive pap test; higher than women in buildings 46% or at CerviCusco (29.2%)<br>More than 90% women in tents or buildings would go to CerviCusco for f/u care	

telemedicine-enhanced lung cancer screening using mobile computed tomography unit with remote artificial intelligence assistance in underserved communities: initial results of a population cohort study in western china	2024	Telemed J E Health	Field and clinic	China	Urban and rural	Individuals, 40-80	Lung cancer	LDCT	Single screening service	Doctors and nurses from West China Hospital conducted recruitment Driver, CT technician, radiologists (interpret CTs) Mobile LDCT scanner Each mobile unit stayed at each site for 2 months	Nurses, radiologists, CT technicians	Operating room witht adjustable scanning bed Command room Interior work space Anterior/lateral entrances	Conducted in mobile unit	West China Hospital	Financial sustainability Maintenance costs Internet connectivity	Lung cancer detected Treatment outcomes Follow-up	19517 participants were screened 2.68% had high-risk pulmonary nodules 0.55% were diagnosed with lung cancer after 1 year 62.98% of those with high-risk nodules received timely hospital treatment 1.69% received treatment in	Short observational period (feasibility assessment) No cost-effectiveness evaluation

																hospitals 0.86% were under regular check up for dx	
increasing cervical cancer screening in a hispanic migrant farmworker community through faith-based clinical outreach	2011	J Low Genit Tract Dis	Field	United states	Central Florida (rural)	Women, low-income, hispanic migrant farmworkers	Cervical cancer	Pap test	Single screening service	Faith-based clinic volunteer medical professionals and "qualified staff" Required to be low-income or uninsured to be eligible	Not specified	Not specified	Conducted in mobile unit	Catholic Mobile Medical Services Moffitt Cancer Center	Not specified	Adherence to screening	Women who have lived in the US for more than 5 years were more adherent with cervical cancer screening recommendations compared to women who have lived in the US for less than 5	Selection bias Incomplete follow-up data Limited generalizability

																	years (p = 0.05) Married women were more likely to be adherent than unmarried women (p = 0.02)	
effect of a mobile unit on changes in knowledge and use of cervical cancer screening among rural thai women	1995	Int J Epidemiol	Field	Thailand	Mae Sot District (rural)	Women, 18-65, not undergone hysterectomy	Cervical cancer	Pap test	Single screening service	Health education and pap tests 54 rural villages in Thailand Health educators, public health nurses, health center workers, physicians Education provided through	Trained public health nurses, midwives, nurses, supervising physician	Not specified	Conducted in health centers/schools	Department of Community and Social Medicine, Mae Sot General Hospital	Lack of prior knowledge Older women tended to participate less	Knowledge of pap test Screening prevalence	6816/16705 women were screened (40.8%) Knowledge of Pap test increased from 20.8% (before) to 57.3% (after) Screening prevalence	Not specified

										radio, leaflets, group lectures, discussions Pap smears collected at village health centers or primary schools							increase from 19.9% to 58.1% Among those who knew about pap test, 92.5% had been screened previously	
program for prostate cancer screening using a mobile unit: results from brazil	2010	urology	Field and clinic	Brazil	Rural and urban	Men, ≥45, limited access to healthcare	Prostate cancer	Digital rectal exam; Serum total and free prostate-specific antigen tests	Single screening service	Mobile unit visited 231 municipalities General physician conducting prostate screening; urologists for follow-up Men w/ abnormal	General physicians trained in prostate cancer screening Urologists	Not specified	Conducted in mobile unit	Local health secretaries Publicity	Limited repeat screenings	Cancers detected PPV Treatment	17571 men screened 3.7% diagnosed w/ prostate cancer 39.6% PPV Among diagnosed cases, Stage 1 = 76.3%	Low number of repeat screenings Cities only visited once Loss to follow-up Noncompliance

										results recalled for further assessment and bx at the hospital							Stage 2 = 17.0%<br>Stage 3 = 6.1%<br>Stage 4 = 0.5%<br>Treatment for dx cases<br>- Observation = 4.7%<br>- Radical prostatectomy = 34.3%<br>- External beam radiotherapy = 50.0%<br>- Androgen deprivation therapy = 11.0%	

baseline results of the west london lung cancer screening pilot study – impact of mobile scanners and dual risk model utilisation	2020	lung cancer	Field and clinic	United Kingdom	West London (urban)	Ever-smokers, 60-75	Lung cancer	LDCT	Single screening service	Hospital-based site and supermarket car parks housed Participants received Lung Health Check, consultation, spirometry, calculation of PLCOM 2012 and LLPv2 Scans interpreted by thoracic radiologists	Radiologists had ≥8 years of experience	Mobile unit contained GE Optima CT660 scanner	Conducted in mobile unit	NHS England and RM Partners	Distance to mobile site longer than distance to hospital	Lung cancer detection Cancer stage Intent to treat	1145 participants total 2.5% detection rate Cancer stage<br>- Stage I = 58.6%<br>- Stage II = 3.4%<br>- Stage III = 20.7%<br>- Stage IV = 17.2%<br><br>For the 24 participants without metastasis, 70.8% were scheduled for tx with curative intent	lower uptake compared to other UK pilot studies Not directly comparable populations between mobile unit and hospital

response to a mobile cervical screening unit in an industrial area of england	1976	Public health	Field and clinic	United Kingdom	Salford (urban)	Women, 35+	Cervical cancer	Pap smear	Single screening service	WNCCC caravan brought to salford for cervical cancer screening Caravan stationed for 3 weeks	Not specified	Not specified	Conducted in mobile unit	Women's National Cancer Control Campaign	Older women more reluctant to participate	Abnormal smear detection rate in mobile unit Uptake	1549 women screened 6.6 per 1000 abnormal smear detection rate Women 35+ underrepresented in caravan screening - 58.2% caravan - 67.7% Salford clinics	Low yield of abnormal Pap smears Caravan visit displace clinic attendance Lack of follow-up
real-time mobile teledermoscopy for skin cancer screening	2016	Journal of the European Academy of Dermatology and	Field	France	Rural	Individuals, 18+	Skin cancer	Real-time mobile teledermoscopy	Single screening service	15 screening centers 21 trained occupational physicians iPhone 4	Occupational physicians trained via 1-day dermatology course	Not specified	Conducted in mobile unit	Mutualité Sociale Agricole Syndicat National des Derma	Image quality Need real-time dermatologist	Lesions identified Cancer cases Follow-up	289 patients screened 199 (69%) had suspicious	Conducted on a single day Limited sample size No comparison group

targeting an agricultural population: an experiment on 289 patients in france		Venereology								w/ Handyscope V2 to take photos experienced dermatologists provided real time diagnosis	Dermatologists had extensive experience in dermoscopy			to-Vénérologues de France	availability		lesions<br>53% did not require follow-up<br>12 patients had suspected melanomas<br>1 diagnosed case of melanoma<br>11/12 patients were seen by dermatologist within 10 days	
low-cost screening mammography: results	1993	Southern Medical Journal	Field and clinic	United States	Lynchburg (urban)	Women, 35+	Breast cancer	Mammography	Single screening ser	Mammography Center opened as a separate screening facility	Radiolgoists	Not specified	Conducted in mobile unit	The Mammography Center	Self-referral<br>Expensive screening	Screening results	Cumulative recall rate = 6%<br>14 cases in	Not specified

of 21141 consecutive examinations in a community program									vice	from hospitals; mobile mammography unit added as well. Two-view medial-lateral oblique and cranial caudad study using phototiming, low kilovolt peak technique, single emulsion film, and dedicated extended processing<br>$45 cost<br>Patients with normal findings are recalled after 1							group aged 40-49<br>Average age found to have cancer = 61.5<br>98 cancers detected total	

										year Radiologists interpret scans							
compliance with breast cancer screening achieved by the aylesbury vale mobile service (1984-1988)	1990	Journal of Public Health	Field	United Kingdom	Aylesbury Vale	Women, 45-64	Breast cancer	Mammography	Single screening service	Mobile van included a waiting area, examination room, x-ray room Nurses conducted exams and x-rays People recruited via letters and reminders	Not specified	Converted Trimoco van Waiting room, exam room, X-ray room	Conducted in mobile unit	Department of Community Medicine and General Practice, Radcliffe Infirmary	Outdated population registers	Compliance	73.7% women who were invited, attended screening 91.5% compliance among previously screened women 67.6% compliance among unscreened women 8.1% additional attendance after remin	Difficulty tracking non-responders Potential over/underestimation 2/2 unknown invitation receive Low compliance in older age groups

																	der letters sent out	
